# Metabolic and Phenotypic Differences between Mice Producing a Werner Syndrome Helicase Mutant Protein and Wrn Null Mice

**DOI:** 10.1371/journal.pone.0140292

**Published:** 2015-10-08

**Authors:** Lucie Aumailley, Chantal Garand, Marie Julie Dubois, F. Brad Johnson, André Marette, Michel Lebel

**Affiliations:** 1 Centre de Recherche du CHU de Québec, Faculty of Medicine, Laval University, Quebec City, Quebec, Canada; 2 Quebec Heart and Lung Institute, Faculty of Medicine, Laval University, Quebec City, Quebec, Canada; 3 Department of Pathology and Laboratory Medicine, University of Pennsylvania, Philadelphia, Pennsylvania, United States of America; University of Texas Health Science Center at San Antonio, UNITED STATES

## Abstract

Werner syndrome (WS) is a premature aging disorder caused by mutations in a RecQ-family DNA helicase, WRN. Mice lacking part of the helicase domain of the WRN orthologue exhibit many phenotypic features of WS, including metabolic abnormalities and a shorter mean life span. In contrast, mice lacking the entire Wrn protein (i.e. Wrn null mice) do not exhibit a premature aging phenotype. In this study, we used a targeted mass spectrometry-based metabolomic approach to identify serum metabolites that are differentially altered in young Wrn helicase mutant and Wrn null mice. An antibody-based quantification of 43 serum cytokines and markers of cardiovascular disease risk complemented this study. We found that Wrn helicase mutants exhibited elevated and decreased levels, respectively, of the anti-inflammatory cytokine IL-10 and the pro-inflammatory cytokine IL-18. Wrn helicase mutants also exhibited an increase in serum hydroxyproline and plasminogen activator inhibitor-1, markers of extracellular matrix remodeling of the vascular system and inflammation in aging. We also observed an abnormal increase in the ratio of very long chain to short chain lysophosphatidylcholines in the Wrn helicase mutants underlying a peroxisome perturbation in these mice. Remarkably, the Wrn mutant helicase protein was mislocalized to the endoplasmic reticulum and the peroxisomal fractions in liver tissues. Additional analyses with mouse embryonic fibroblasts indicated a severe defect of the autophagy flux in cells derived from Wrn helicase mutants compared to wild type and Wrn null animals. These results indicate that the deleterious effects of the helicase-deficient Wrn protein are mediated by the dysfunction of several cellular organelles.

## Introduction

Werner syndrome (WS) is a human autosomal recessive disorder characterized by genomic instability and the premature onset of a number of age-related diseases [1–4]. The defective enzyme responsible for WS possesses a 3′-5′ exonuclease activity in addition to a 3′-5′ DNA helicase activity [5, 6] and is involved in DNA repair, replication, transcription, and telomere maintenance [7–11]. More than seventy distinct mutations inactivating the WRN protein have been described in WS patients to date [12, 13]. These mutations include missense and nonsense substitutions, frame shifts and premature translation termination mutations, deletions and insertions. All these mutations are believed to disrupt the normal function of the protein or to cause a truncation of the protein such that it cannot localize to the nucleus, the normal site of WRN protein action [9]. We previously generated a mouse model with a deletion of part of the helicase domain of the murine WRN homologue (referred as *Wrn* ^*Δhel/Δhel*^ mice hereafter) [14] that recapitulates many of the WS phenotypes, including an abnormal hyaluronic acid excretion, higher reactive oxygen species (ROS) levels, dyslipidemia, increased genomic instability, and cancer incidence [15, 16]. Overall, such mutant mice have a 10–15% decreased of their mean life span [17, 18]. Of relevance, increased oxidative stress has been described for WS subjects in addition to abnormal metabolic phenotypes [19–21]. In addition to the metabolic abnormalities found in *Wrn* ^*Δhel/Δhel*^ mice, microscopic analysis of tissues revealed three other major changes; increased levels of visceral fat tissues, defenestration of liver sinusoidal endothelial cells (an increasingly recognized morphological change associated with normal aging [22]), liver steatosis [23], and aortic stenosis [24] followed by cardiac fibrosis [16]. *Wrn* ^*Δhel/Δhel*^ mice synthesize a stable mutant protein that can be detected by standard immunoblotting techniques and which has a smaller size consistent with the number of deleted amino acids [14].

A second mutant mouse model for WS was generated in the same period [25]. No Wrn protein is detected in cells or tissues of these mutant mice (referred as *Wrn* ^*-/-*^ or *Wrn* null mice hereafter). These mice live beyond two years of age without any obvious phenotype [25]. These mice, however, develop a hyperinsulinemia and insulin resistance when fed with a diabetogenic diet [26]. The reason for the differences between the phenotypes of *Wrn* ^*Δhel/Δhel*^ and *Wrn* ^*-/-*^ mice is unknown. In this study, we compared the metabolic profile of these mutant mice using a targeted mass spectrometric analysis. We measured amino acids, biogenic amines, acylcarnitines, lysophosphatidylcholines, glycerophosphatidylcholines, sphingomyelines, and prostaglandins in the serum of wild type (WT), *Wrn* ^*Δhel/Δhel*^, and *Wrn* ^*-/-*^ mice at four-five months of age. So as to avoid indirect effects caused by any pathology, we quantified these metabolites at an age at which no obvious microscopic anomalies could be detected in the *Wrn* ^*Δhel/Δhel*^ mutant mice, such as liver endothelial defenestration, visceral fat accumulation, or abnormal increased oxidative stress [18, 23]. We also examined the levels of several inflammatory cytokines, metabolic hormones, and markers of cardiovascular diseases in these animals. Interestingly, we found that the Wrn helicase mutant protein is not found in the nucleus but rather in organelles that affect the metabolite profile of *Wrn* ^*Δhel/Δhel*^ mice.

## Materials and Methods

### Animal model

Mice lacking part of the helicase domain of the *Wrn* gene were generated by homologous recombination, as described previously [14]. In the process, 121 amino acid residues of the Wrn protein were deleted (amino acids 710 to 831). The generation of *Wrn* ^*-/-*^ mice has been reported [25]. This study was performed on WT, *Wrn* ^*Δhel/Δhel*^, and *Wrn* ^*-/-*^ homozygous animals backcrossed on the C57BL/6N genetic background (for a minimum of seven generations). This study was carried out in strict accordance with the recommendations in the Guide for the Care and Use of Laboratory Animals of the Canadian Council on Animal Care in science. The protocol was approved by the Committee on the Ethics and Protection of Animal of Laval University (Permit Number: 2014029). Mice were housed in cages (containing a top filter) at 22 ± 2°C with 40%–50% humidity and a 12-h light–dark cycle (light cycle: 06:00–18:00 hours) in the Centre de Recherche de l’Hôtel-Dieu de Québec animal facility. All mice were fed *ad libitum* with Teklad Global (Madison, WI) 18% protein rodent diet (5% fat). Euthanasia was performed by treating mice with 3% isoflurane (general anesthesia) followed by cervical dislocation.

### Blood profile

Blood (50 μL) was collected by puncture of the leg’s saphenous vein at the age of four months in EDTA containing tubes (Sarsted Inc., Newton, NC). Blood parameters were measured in a VetScan HM5c (Abaxis, Union City, CA). Parameters included white blood cell count, lymphocyte count, monocyte count, neutrophil count, platelet count, red blood cell count, mean cell volume, hemoglobin, and % hematocrit.

### Serum collection for analysis

Blood was harvested at 10:00 am by cardiac puncture and exsanguination under anesthesia at the age of four months. Blood was allowed to clot for one hour at 4°C and spun on a bench top centrifuge at 16,000 x g for 15 min. Serum was collected and frozen at -80°C until execution of the analyses. Ascorbic acid in serum was measured with the ferric reducing ascorbate assay kit from BioVision Research Products (Mountain View, CA, USA). Serum levels of glutathione (GSH) were quantified with the ApoGSH Glutathione Detection Kit from Bio Vision (Mountain View, CA). Fluorescence was measured with a Fluoroskan Ascent fluorescence spectrophotometer (Thermo Electron Inc., Milford, MA). Quantification of reactive oxygen species was performed as described previously [16].

### Metabolite measurements

Metabolite measurements were performed by the BIOCRATES Life Sciences metabolomic services (BIOCRATES Life Sciences AG, Innsbruck, Austria). Briefly, Biocrates’ commercially available kit plates were used for the quantification of amino acids, acylcarnitines, sphingomyelins, phosphatidylcholines, hexoses, and biogenic amines. The fully automated assay was based on PITC (phenylisothiocyanate)-derivatization in the presence of internal standards followed by FIA-MS/MS (acylcarnitines, lipids, and hexose) and LC/MS (amino acids, biogenic amines) using an AB SCIEX 4000 QTrap® mass spectrometer (AB SCIEX, Darmstadt, Germany) with electro spray ionization. The experimental metabolomics measurement technique has been previously described [27]. Eicosanoids and other oxidized polyunsaturated fatty acids were extracted from samples with aqueous acetonitrile that contained deuterated internal standards. The metabolites were determined by HPLC-tandem mass spectrometry (LC-MS/MS) with Multiple Reaction Monitoring (MRM) in negative mode using a SCIEX API 4000 QTrap mass spectrometer with electro spray ionization. The LC-MS/MS method used for the analytical determination of eicosanoids has been published [28]. Accuracy of the measurements (determined with the accuracy of the calibrators) was in the normal range of the method (deviations from target ≤ 20%) for all analytes. In total, 203 different metabolites were measured. Metabolomics data set contains 21 amino acids, 19 biogenic amines, one hexose (H1), free carnitine (C0), 40 acylcarnitines (C*x*:*y*), hydroxylacylcarnitines (C(OH)*x*:*y*), and dicarboxylacylcarnitines (C*x*:*y*-DC), 15 sphingomyelins (SM*x*:*y*) and *N*-hydroxylacyloylsphingosylphosphocholine (SM (OH)*x*:*y*), 77 phosphatidylcholines (PC, aa = diacyl, ae = acyl-alkyl), 14 lysophosphatidylcholines, and 17 eicosanoid acids and prostaglandins. Lipid side chain composition is abbreviated as C*x*:*y*, where *x* denotes the number of carbons in the side chain and *y* the number of double bonds. For example, “PC ae C30:1” denotes an acyl-alkyl phosphatidylcholine with 30 carbons in the two fatty acid side chains and a single double bond in one of them [27]. Full biochemical names are provided in the S1 Table. The precise position of the double bonds and the distribution of the carbon atoms in different fatty acid side chains cannot be determined with this technology.

### Cytokine measurements

The following cytokines were assessed in the serum (diluted 1:4) using the multiplex kit from Bio-Rad Laboratories Canada Ltd. (catalogue number MD000000EL; Mississauga, ON, Canada): IL-15, IL18, LIF, M-CSF, MIG, MIP-2, PDGF-BB, VEGF, and FGF basic. The following cytokines were assessed in the serum (diluted 1:4) using the multiplex kit from Bio-Rad (catalogue number MD60009RDPD): IL-1α, IL-1β, IL-2, IL3, IL-5, IL-6, IL-10, IL-12(p40), IL-12(p70), IL-13, IL-17, KC, MCP-1, MIP-1α, MIP-1β, TNF-α, RANTES, Eotaxin, G-CSF, GM-CSF, and IFN-γ. The metabolic hormones C-peptide 2, GIP, leptin, and resistin were assessed in the serum using the Milliplex Mouse Metabolic Magnetic Bead Panel (catalogue number MMHMAG-44K) from EMD Millipore Corp. (Billerica, MA, USA). The following cardiovascular risk factors were assessed in the serum (diluted 1:20) using the Milliplex Mouse CVD Panel 1 kit from EMD Millipore Corp. (cat. MCVD1MAG-77K): soluble E-selectin, P-selectin, ICAM-1, Pecam-1, proMMP-9, thrombomodulin, and total PAI-1. Full biological names and raw data are provided in the S2 Table. All measurements were performed on a Bio-Plex® 200 system (with Bio-Plex Manager™ software version 6.0) from Bio-Rad Laboratories Canada Ltd. (Mississauga, ON, Canada). Mouse erythropoietin was measured with an ELISA kit from R&D Systems, Inc. (Minneapolis, MN).

### Mouse Embryonic Fibroblasts (MEFs)

Generation and maintenance of embryonic fibroblasts has been described previously [29]. Briefly, healthy 15.5-day-old embryos were minced in 6-well plates and maintained in low glucose DMEM supplemented with 10% of heat-inactivated calf serum at 37°C in an atmosphere of 5% CO2. When indicated, exponentially growing cells or confluent cultures of MEFs were treated three hours with 50 μM chloroquine diphosphate salt (Sigma-Aldrich, Oakville, ON) before protein extraction in CHAPS lysis buffer (30 mM Tris-HCl pH 7.5, 150 mM NaCl, 1% CHAPS). Cells were lysed on ice for 30 minutes with frequent mixing. Protein concentration was determined by the Bradford assay (Bio-Rad, Mississauga, ON, Canada). Nuclear and cytoplasmic extracts from MEFs were obtained using a nuclear/cytosol fractionation kit (BioVision, Milpitas, CA) according to the manufacturer’s protocol.

### Measurements of Reactive oxygen species in MEFs

Cells were washed with PBS, scraped, pelleted, and resuspended in RIPA buffer (50 mM Tris–HCl (pH 7.5), 150 mM NaCl, 1% NP-40, 0.1% SDS, 0.5% sodium deoxycholate, 1mM phenylmethylsulfonylfluoride and complete protease inhibitor cocktail (Roche Applied Science, Indianapolis, IN)). Cell debris were spun down. The lysate (420 μg of proteins in a total volume of 350 μL) was incubated with 10 μg/mL of the dye 2’–7’ dichlorofluorescein diacetate (Sigma-Aldrich Canada Ltd, Oakville, ON) for 1 h at 37°C. This dye is highly fluorescent upon oxidation. As control, RIPA buffer was also incubated with dichlorofluorescein diacetate and 100 μL (120 μg of proteins) of the samples were put into 96-well plates. Fluorescence was measured with a Fluoroskan Ascent fluorescence spectrophotometer (Thermo Electron Inc., Milford, MA). The excitation and emission wavelengths used were 485 and 527 nm, respectively. Background fluorescence was extracted from the dichlorofluorescein value for each sample and the final result was expressed as units of fluorescence per gram of proteins.

### Protein sulfhydryl measurements in MEFs

Cells were washed with PBS, scraped, pelleted, and resuspended in 20 mM sodium phosphate buffer (pH 7.4) containing 140 mM potassium chloride. Cells were lysed on ice for 30 min. Protein sulfhydryls were measured by monitoring the reaction between thiol groups on proteins and the colorimetric reagent 5,5'-dithio-bis-(2-nitrobenzoic acid) (DTNB, Sigma-Aldrich, Oakville, ON) as described before [30].

### Protein extraction from tissues

Weighed tissues were homogenized on ice in lysis buffer (20 mM MOPS pH 7.0, 2 mM EGTA, 5 mM EDTA, 30 mM NaF, 60 mM glycerophosphate pH 7.2, 20 mM sodium pyrophosphate, 1 mM sodium orthovanadate, 1 mM PMSF, 3 mM benzamidine, 5 μM pepstatin A, 10 μM leupeptin, 0.5% Triton X-100, 1 mM DTT). Samples were then incubated in lysis buffer on ice for 15 min followed by an additional 15 min on a rotary device at 4°C. Lysates were sonicated twice for 10 sec on ice to shear nuclear DNA. Samples were centrifuged twice at 130,000 rpm for 15 min at 4°C to remove cell debris. Protein concentration was determined by the Bradford protein assay (Bio-Rad, Mississauga, ON, Canada).

### Fractionation procedures for tissues

Total endoplasmic reticulum (ER) fractions from mice livers were obtained using an endoplasmic reticulum enrichment assay kit (Novus Biologicals, Burlington, ON, Canada) according to the manufacturer's protocol. Samples were frozen at -80°C until western analyses. Peroxisomal fractions from mice livers were obtained using a peroxisome enrichment kit (ThermoFisher Scientific, Waltham, MA) according to the manufacturer's protocol. The peroxisomal fraction was resuspended in IP Buffer (50 mM Tris-HCl pH 8.0, 120 mM NaCl, 0.5% NP-40) and frozen at -80°C when indicated.

### Immunoprecipitation and immunoblotting

All steps were performed on ice or at 4°C. Two PBS washes were carried out prior to the extraction (cell scraping) with 2 mL/plates of lysis buffer [40 mM HEPES pH 7.5, 120 mM NaCl, 0.3% CHAPS, 1 mM EDTA, Complete protease inhibitor cocktail (Roche Applied Science, Indianapolis, IN)]. Immunoprecipitation experiments were performed using Dynabeads magnetic beads covalently coupled with Protein G (Invitrogen, Burlington, ON). The Dynabeads were washed two times with 1 mL of 0.1 M sodium acetate buffer, pH 5.0, coated with 10–15 μg of mouse monoclonal anti-Wrn antibody. The mouse monoclonal antibody was generated by Abmart Inc. (Shanghai, China) antibody service with the peptide PNDDENDSSY representing amino acids 420–429 of the mouse Wrn protein. This antibody can immunoprecipitate the Wrn protein but it cannot be used for western blotting or immunofluorescence techniques. The antibody-coupled Dynabeads were incubated for 1 h at room temperature with 1 mL of PBS containing 1% (w/v) BSA (Sigma-Aldrich, Oakville, ON) to block nonspecific antibody binding sites. The beads were finally washed three times with 1 mL of lysis buffer and added to the protein extract for 4 h incubation with gentle agitation in a cold room. Samples were then washed three times with 2 volumes of lysis buffer for 5 min. Protein complexes were eluted using 150 μL of 2 x Laemmli sample buffer. Proteins were resolved using 4–12% Criterion XT Bis-Tris gradient gel (Bio-Rad, Mississauga, ON, Canada) and then transferred onto 0.2 μm PVDF membrane (EDM Millipore Corp., Temecula, CA). After incubating 1 h with blocking solution (PBS-T containing 5% nonfat milk), the membrane was probed overnight at 4°C with a rabbit polyclonal antibodies against WRN (antibody H-300 from Santa Cruz Biotechnology, Santa Cruz, CA). The latter antibody recognizes both the mouse and human WRN proteins. After washing with PBS-T, species-specific horseradish peroxidase-conjugated secondary antibody was added for 2 h at room temperature (GE Healthcare Limited, Piscataway, NJ). Signals were generated with Western Lightning Chemiluminescence reagent plus kit (GE Healthcare Limited, Piscataway, NJ).

When indicated, immunoblots were probed with the following antibodies: rabbit polyclonal antibodies against calreticulin (ER marker; ab39818) and topoisomerase I (nuclear marker; ab109374) from Abcam (Cambridge, MA); mouse monoclonal antibodies against catalase (C0979) and actin (A5441) from Sigma-Aldrich (Oakville, ON); a mouse monoclonal β-tubulin antibody (E7) provided by Michael Klymkowky (University of Colorado, Boulder, CO); a rabbit polyclonal antibody against glucose-related protein 78 (anti-GRP78) from Proteintech^TM^ (Chicago, IL); rabbit polyclonal antibodies against phospho-inositol-requiring kinase 1α (anti-phosphoIRE1α, NB100-2323) and Light Chain 3 (anti-LC3; NB100-220) from Novus Biologicals (Burlington, ON, Canada); a rabbit monoclonal antibody against inositol-requiring kinase 1α (anti-IRE1 #3294) from Cell Signaling Technology (Beverly, MA).

### Exonuclease and helicase activities of immunoprecipitated Wrn proteins

One confluent 150-mm petri dish of MEFs was lysed in 1mL of a stringent buffer [50 mM Tris-HCl pH 8.0, 150 mM NaCl, 1.0% NP-40, 0.1% SDS, 0.5% sodium deoxycholate, Complete protease inhibitor cocktail (Roche Applied Science, Indianapolis, IN)]. Wrn proteins were immunoprecipitated with 2 μg of an antibody against mouse Wrn and magnetic beads as described above. Immunoprecipitation was carried out for 2 h in a cold room. Beads containing the immune complexes were washed once with 1 mL of a buffer containing 20 mM Tris-HCl pH 8.0, 0.5 M NaCl, 1 mM EDTA, 0.5 mM DTT, 0.5% NP-40, 25% glycerol, 0.2 mM PMSF. Beads were then washed twice with 2 mL of a buffer containing 20 mM Tris-HCl pH 8.0, 150 mM NaCl, 25% glycerol, 0.5% NP-40, 0.05% sodium deoxycholate, 0.005% SDS. Finally, beads were resuspended in 15 μL of buffer (25 mM Tris-HCl pH 8.0, 0.5 mM EDTA, 1 mM DTT, 0.05% NP-40, 25% glycerol). Re-suspended beads containing the immune complexes (0.1 μg/μL of antibody) were diluted as indicated in the figure legends in assay reaction buffer (40 mM Tris-HCl pH 7.4, 4 mM MgCl_2_, 0.1 mg/mL BSA, 5 mM DTT, and 100 nM of splayed arms labeled on one DNA strand) [31]. The reaction was incubated for 20 min at 37°C, and stopped with one-fifth volume of Stop buffer (40% glycerol, 50 mM EDTA, 2% SDS, 3% xylene cyanol and 3% bromophenol blue). Reaction samples were loaded on a native 12% polyacrylamide gel (in TBE buffer) for autoradiography to analyze the DNA helicase activity. For the exonuclease activity, cleaved DNA products were separated on a denaturing polyacrylamide gel (14%, 8 M urea in TBE) and analyzed by autoradiography.

### Immunofluorescence analyses

The mouse Wrn cDNA and the cDNA bearing a deletion of part of the helicase domain were cloned into the eGFP-C3 vector (BD Biosciences Clontech, Palo Alto, CA) to generate the pmGFPWrn and pmGFPWrn^Δhel^ constructs. The mouse WT Wrn (Myc-DDK-tagged) was obtained from OriGene Technologies, Inc. (Rockville, MD) (called pmDDKWrn hereafter). Part of the cDNA containing the helicase deletion was swapped for the WT sequence to generate the pmDDKWrn^Δhel^ construct. MEFs were trypsinized, pelleted (200 x *g* at room temperature for 10 min) and 1.0 x10^-6^ cells were transfected with 5 μg of one of the four plasmids (pmGFPWrn, pmGFPWrn^Δhel^, pmDDKWrn and pmDDKWrn^Δhel^) using a nucleofector® kit (ESBE Scientific, Markham, ON). Transfected cells were seeded on sterile cover slips and allowed to grow overnight. Cells were then fixed with pre-warmed 4% paraformaldehyde (Fisher, Fair Lawn, NJ) in PBS for 20 min at 37°C and permeabilized with 0.15% Triton (USB, Salem, MA) in PBS for 10 min at room temperature. Cells were blocked by incubation with 3% bovine serum albumin (Fisher Scientific, Fair Lawn, NJ) in PBS for 1 hour at room temperature, followed by an overnight incubation at 4°C with the indicated primary antibodies. The rabbit monoclonal antibodies against PDI (C81H6; ER marker) and AIF (D39D2; mitochondrial marker) from the organelle localization IF antibody sampler kit (Cell Signaling Technology, Beverly, MA) were used. The rabbit polyclonal antibodies against Lamp1 (ab24170; lysosomal marker) and catalase (ab15834; perosisomal marker) were purchased from Abcam (Cambridge, MA). The mouse monoclonal antibody against the DDK tag was purchased from Origene (Rockville, MD). Finally, cells were incubated with the appropriate fluorescence-coupled secondary antibody antibodies (a polyclonal Alexa Fluor® 594 or Alexa Fluor® 488 conjugated anti-rabbit IgG secondary antibody or a polyclonal Alexa Fluor® 594 conjugated anti-mouse IgG from Life Technologies, Burlington, ON) for 1 hour at room temperature. Confocal images were taken with a FV1000 laser-scanning confocal microscope equipped with a 60 x 1.4 NA oil-immersion lens and Fluoview FV10 asw 3.1 software (Olympus). Images were processed using ImageJ version 1.48j. The quantitative co-localization analysis was performed using the JACoP (Just Another Co-localization Plugin) plugin for ImageJ. Images from 3–7 transfected cells expressing the fluorescent or tagged WT or mutant Wrn protein were analyzed for each marker and the Pearson's coefficient was scored [32]. A linear equation describing the relationship between the intensities in two images was calculated by linear regression [33]. The Pearson's coefficient provided an estimate of the goodness of this approximation. ImageJ was also used to create line profiles of the merged images.

### Statistical analyses

One-way ANOVA followed by Tukey’s HSD (honest significant difference) Test for post-ANOVA pair-wise comparisons were performed for all serum metabolites and cytokines. One Way Analysis of Variance was performed for body weight gain. Differences between cohorts were considered significant at values of *P* < 0.05. All tests were calculated using the http://faculty.vassar.edu/lowry/ank3.html website.

## Results

### Life span and body weight of *Wrn* mutant mice

**Fig 1:**
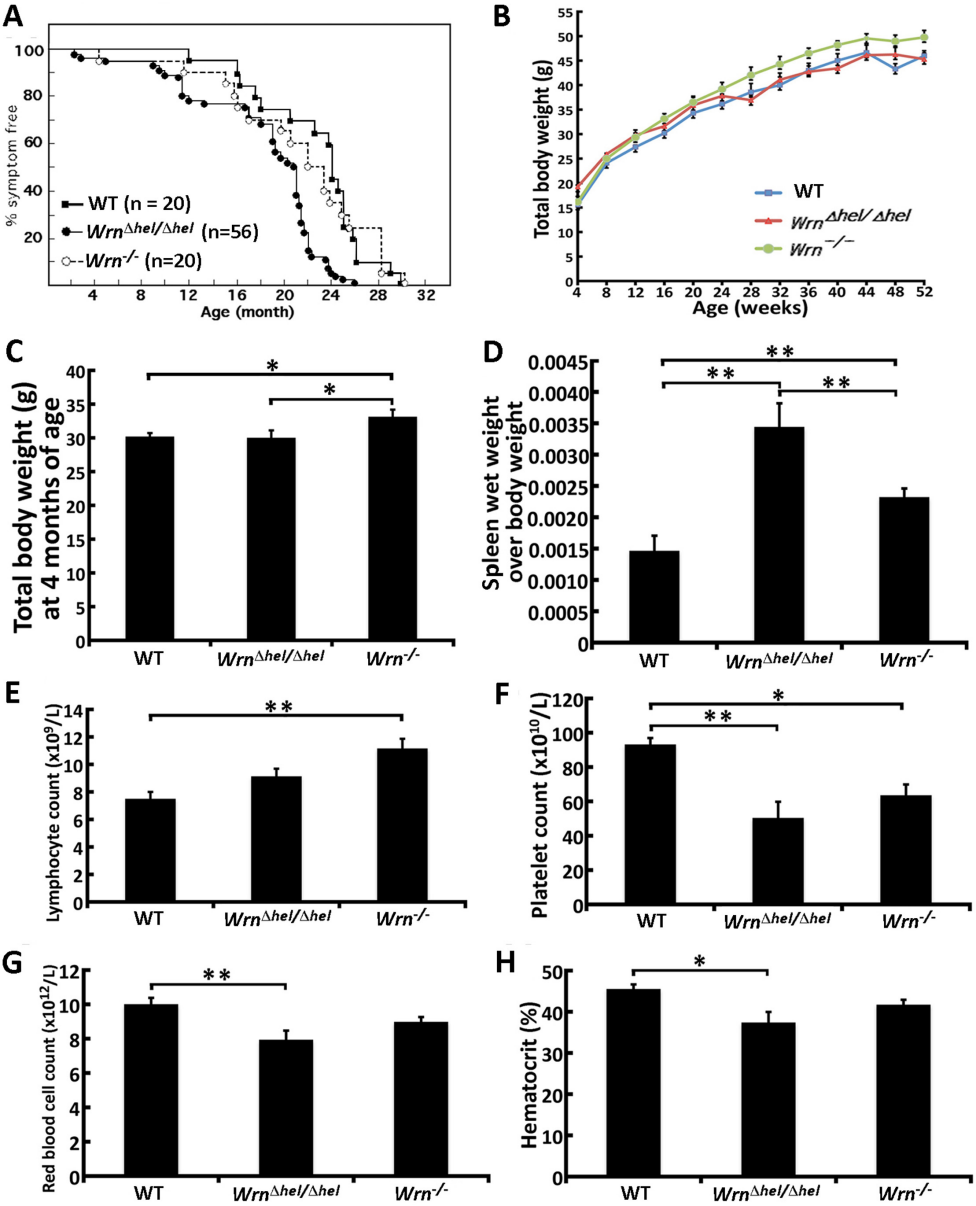
Phenotypic traits of *Wrn* ^*Δhel/Δhel*^ and *Wrn* ^*-/-*^ mice. (A) Percentage of disease-free animals with age. The number of animals in each group is indicated (50% males + 50% females). (Log rank tests: WT *vs*. *Wrn* ^*Δhel/Δhel*^ mice, *P* = 6.8x10^-5^; WT *vs*. *Wrn* ^*-/-*^ mice, *P* = 0.001; *Wrn* ^*Δhel/Δhel*^ *vs*. *Wrn* ^*-/-*^ mice, *P* = 0.955). (B) Measures of males total body weight from the age of 4 to 52 weeks (n = 6–19 males). (C) Total body weight at four months of age (n = 6–10 males). (D) Ratio of spleen wet weight over total body weight at four months of age (n = 6 males). (E) Lymphocyte blood count at four months of age (n = 5 males). (F) Platelet blood count at four months of age (n = 5 males). (G) Red blood cell count at four months of age (n = 5 males). (H) % hematocrit at four months of age (n = 5 males). Bars in all the graphs represent SEM.

We investigated the effect of different mutations in the mouse *Wrn* gene on mean life span. (Each cohort was composed of 50% females to obtain this measure.) The mean life span of *Wrn* ^*Δhel/Δhel*^ mice was ~22% lower (18.7 months) than the mean life span of WT animals (22.8 months) (log rank test: *P* = 6.8x10^-5^). The oldest *Wrn* ^*Δhel/Δhel*^ mouse died at 26 months of age, while the oldest WT mouse died at 30 months of age (Fig 1A). The mean life span of *Wrn* ^*-/-*^ mice was 21.4 months. The oldest *Wrn* ^*-/-*^ mouse died at 30 months of age. The log rank test showed no significant difference in life span between the WT and *Wrn* ^*-/-*^ mice (log rank test: *P* = 0.955), consistent with previous findings [25]. The difference in life span between *Wrn* ^*Δhel/Δhel*^ and *Wrn* ^*-/-*^ mice, in return, was significant (log rank test: *P* = 0.001). The age-associated symptoms have already been described for WT and *Wrn* ^*Δhel/Δhel*^ mice [15, 16]. Pathologies in aged *Wrn* ^*-/-*^ mice included maxillary gland hyperplasia, myeloid hyperplasia or leukemia, lymphoma, thymoma, abdominal ascites, liver steatosis, hepatocarcinoma, prostatic hyperplasia, mammary carcinoma, and ovary cistadenoma. Many *Wrn* ^*-/-*^ individuals exhibited two or more pathologies. Aged WT mice exhibited similar phenotypes but at different frequencies (Table 1). Thus, although these cohorts were small, the incidences of pathologies were different between aged WT and *Wrn* ^*-/-*^ mice.

**Table 1:** Incidence of each phenotype in old wild type and *Wrn* ^*-/-*^ mice.

Symptoms	% wild type (n = 20)	% of *Wrn* ^*-/-*^ mice (n = 20)
maxillary gland hyperplasia	5%	5%
myeloid hyperplasia or leukemia	20%	30%
lymphoma	15%	25%
thymoma	0%	5%
abdominal ascites	5%	10%
liver steatosis	5%	10%
hepatocarcinoma	20%	10%
prostatic hyperplasia	0%	10% of males
mammary carcinoma	0%	10% of females
ovary cistadenoma	30% of females	10% of females
bladder occlusions	20% of males	0%

We also followed the total body weight of mice during the first year of life. More specifically, the weight of each male was measured every four weeks. As indicated in Fig 1B, on average the weight of *Wrn* ^*-/-*^ males was 5–13% heavier than WT or *Wrn* ^*Δhel/Δhel*^ males. WT, *Wrn* ^*Δhel/Δhel*^, and *Wrn* ^*-/-*^ males total body weight gain during their first year of life was 29.2 g, 26.0 g, and 33.6 g, respectively (S1A Fig). The difference in gain weight was significantly larger for the *Wrn* ^*-/-*^ males compared to WT and *Wrn* ^*Δhel/Δhel*^ males (one-way ANOVA *P*-value < 0.05). Fig 1C shows that four-month old *Wrn* ^*-/-*^ males were ~10% bigger than both WT and *Wrn* ^*Δhel/Δhel*^ males. Nevertheless, food and water intake at four month of age was not significantly different between cohorts (S1B and S1C Fig).

We first weighed different organs to get a ratio of organ wet-weight over total body weight for each mouse at the age of four months. The spleen was the only organ that showed a significant increase in wet weight in *Wrn* ^*Δhel/Δhel*^ mice compared to both WT and *Wrn* ^*-/-*^ animals (Fig 1D). The spleen of *Wrn* ^*-/-*^ animals was, on average, significantly bigger than that of WT mice but significantly smaller than that of *Wrn* ^*Δhel/Δhel*^ mice. In contrast, the wet weight of visceral fat, heart, kidney, liver, and lungs were not significantly different between the genotypes (S1D–S1H Fig). Based on this information, we determined the blood profile of these animals. Lymphocyte count was increased in *Wrn* ^*Δhel/Δhel*^ and *Wrn* ^*-/-*^ mice. However, only *Wrn* ^*-/-*^ mice showed a significant increased compared to WT animals (Fig 1E). The platelet count, in return, was significantly decreased in both *Wrn* ^*Δhel/Δhel*^ and *Wrn* ^*-/-*^ mice compared to WT animals (Fig 1F). Finally, only *Wrn* ^*Δhel/Δhel*^ mice showed a significant decrease in red blood cell count and hematocrit (~20%) compared to WT animals (Fig 1G and 1H). However, we have no evidence of bone marrow dysfunction in *Wrn* ^*Δhel/Δhel*^ mice, since bone marrow progenitor counts were not significantly different between *Wrn* ^*Δhel/Δhel*^ and WT animals (data not shown). We also measured the levels of serum erythropoietin in each cohort and noticed an increase in serum erythropoietin in both *Wrn* ^*Δhel/Δhel*^ and *Wrn* ^*-/-*^ mice compared to WT animals but it was not statistically significant (S1I Fig).

### Inflammatory cytokines and metabolic hormones in *Wrn* mutant mice

**Fig 2:**
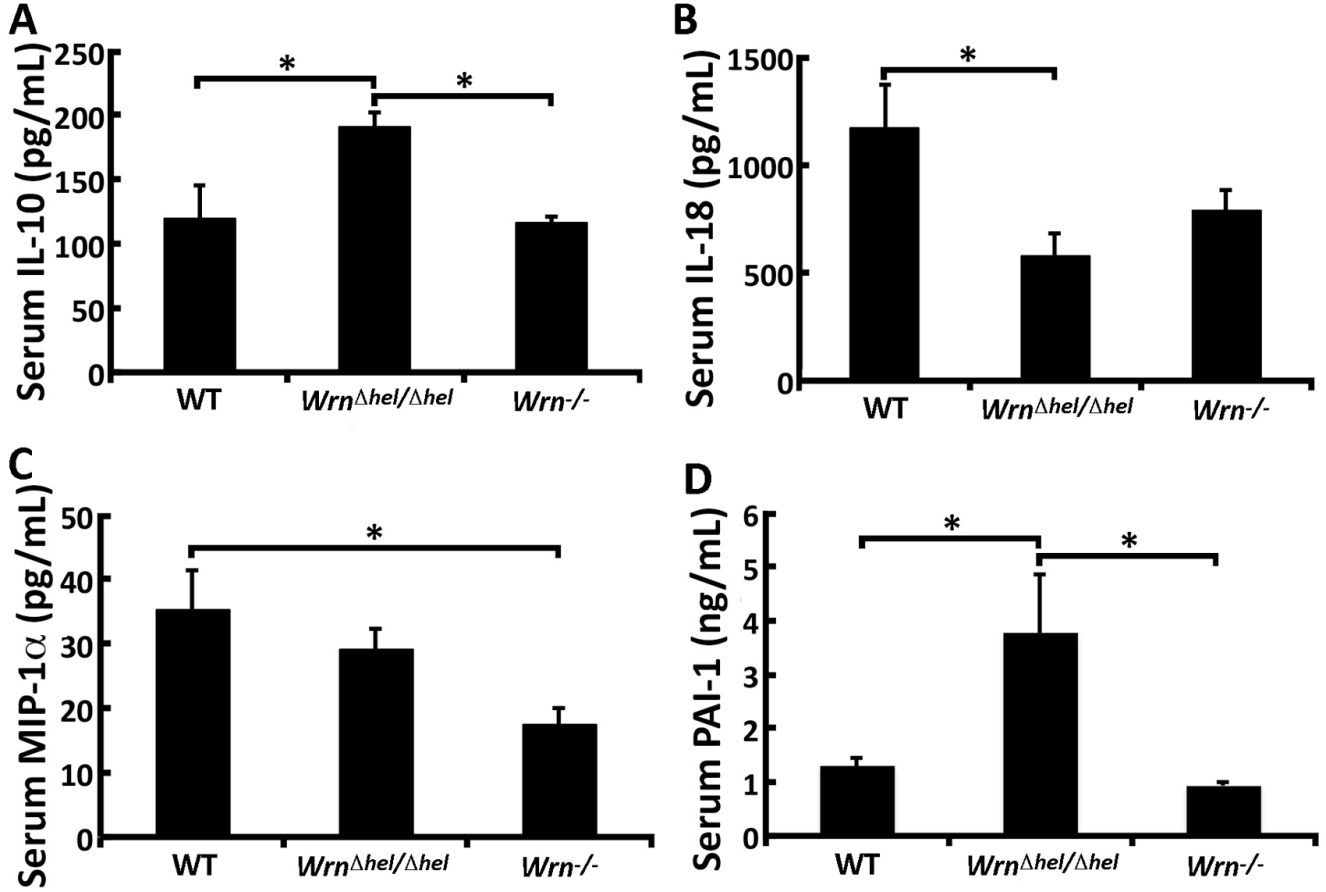
Serum levels of three serum cytokines and one cardiovascular risk factor significantly altered in *Wrn* ^*Δhel/Δhel*^ and *Wrn* ^*-/-*^ mice. (A) IL-10; (B) IL-18; (C) MIP-1α; (D) PAI-1. Tukey post ANOVA test *P*-values are shown (*P < 0.05 and **P < 0.01). Bars in all histograms represent SD. The number of mice in each cohort is indicated in the S2 Table for each serum cytokine.

Since the spleen weight was increased in *Wrn* mutant mice, we next measured the levels of 32 immune cytokines in the serum of our different mouse cohorts. We also quantified the serum level of four metabolic hormones and seven markers of cardiovascular disease risk. Serum cytokine concentrations in animals of each group are shown in the S2 Table. Only three cytokines and the factor PAI-1 (Plasminogen Activator Inhibitor-1) were altered by at least 1.5-fold in one of our groups with a *P*-value < 0.05 (ANOVA in S2 Table). As indicated in Fig 2A, serum IL-10 was significantly increased in *Wrn* ^*Δhel/Δhel*^ mice compared to both WT and *Wrn* ^*-/-*^ mice. IL-18 was significantly decreased in *Wrn* ^*Δhel/Δhel*^ mice compared to WT animals (Fig 2B), while MIP-1α was significantly decreased in *Wrn* ^*-/-*^ mice compared to WT animals (Fig 2C). Finally, serum PAI-1 (Plasminogen Activator Inhibitor-1) was significantly increased in *Wrn* ^*Δhel/Δhel*^ mice compared to both WT and *Wrn* ^*-/-*^ mice (Fig 2D). These results indicate that the helicase mutant *Wrn* ^*Δhel/Δhel*^ mice exhibited an increase in serum IL-10 and PAI-1 that was not found in *Wrn* null animals.

### Metabolic profile of *Wrn* mutant mice

**Fig 3:**
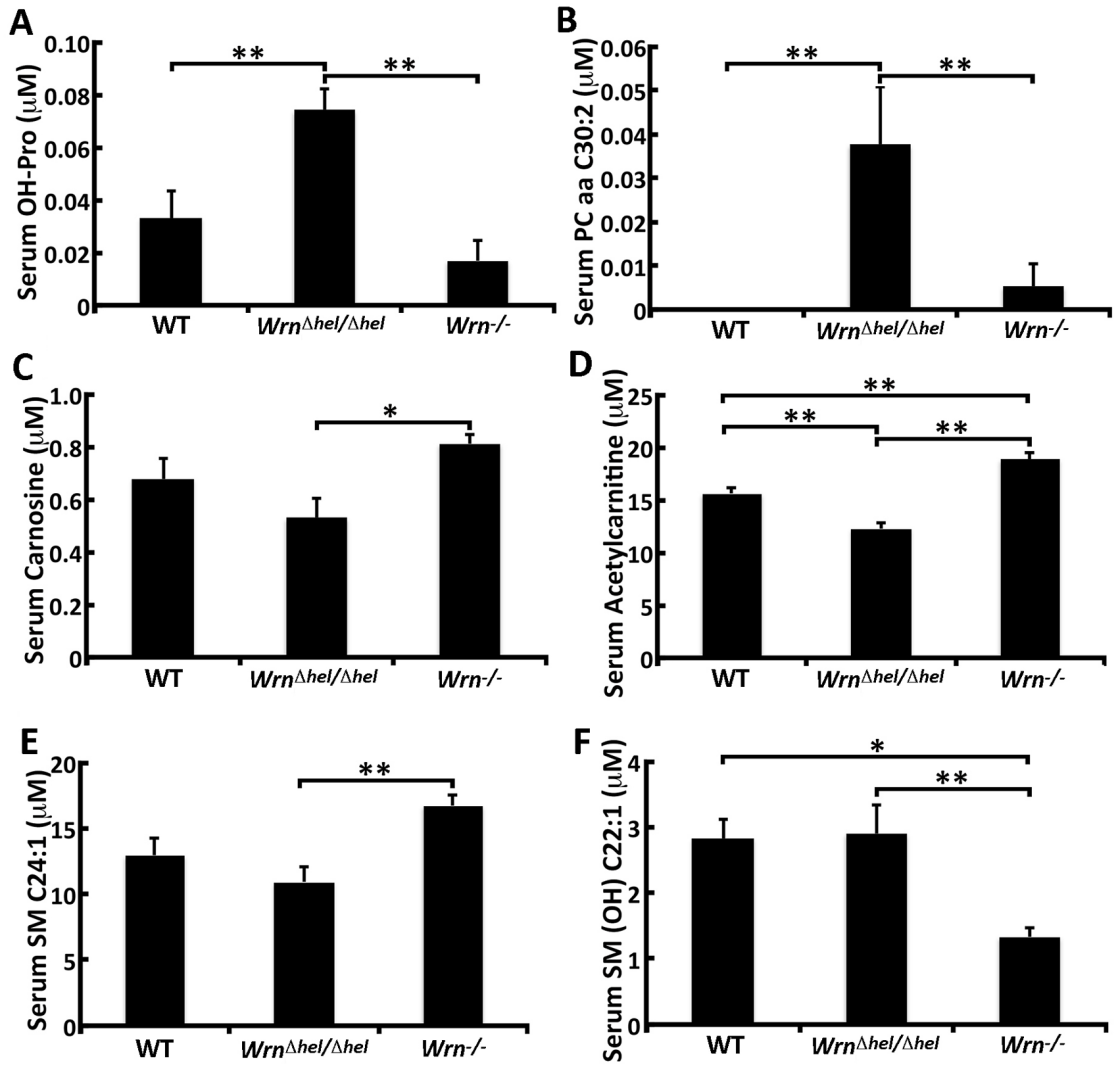
Serum levels of six serum metabolites significantly altered between *Wrn* ^*Δhel/Δhel*^ and *Wrn* ^*-/-*^ mice. (A) Hydroxyproline (OH-Pro); (B) Phosphatidylcholine PC aa C30:2; (C) Carnosine; (D) Acetylcarnitine; (E) Sphingomyelin SM C24:1; (F) Sphingomyelin SM (OH) C22:1. Tukey post ANOVA test *P*-values are shown (*P < 0.05 and **P < 0.01). Bars in all histograms represent SD. N = 6 males for each cohort.

As metabolite disturbances can lead to an inflammatory response or changes in cardiovascular risk factors, we quantified the serum levels of 203 metabolites in our different mouse cohorts. ANOVA tests indicated alteration in the serum levels of 70 significant metabolites between our groups of mice. However, if we focus only on metabolites exhibiting at least a 1.5-fold difference between groups of mice, we see 29 metabolites with significant changes (S3 Table). As indicated in Fig 3A, serum hydroxyproline (OH-Pro) was significantly increased in *Wrn* ^*Δhel/Δhel*^ mice compared to both WT and *Wrn* ^*-/-*^ mice. Similarly, the phosphatidylcholine PC aa C30:2 was significantly increased in *Wrn* ^*Δhel/Δhel*^ mice compared to both WT and *Wrn* ^*-/-*^ mice (Fig 3B). Serum carnosine was significantly increased in *Wrn* ^*-/-*^ mice compared to *Wrn* ^*Δhel/Δhel*^ mice (Fig 3C) and tended to be higher than WT animals. Serum acetylcarnitine was significantly reduced in *Wrn* ^*Δhel/Δhel*^ mice compared to both WT and *Wrn* ^*-/-*^ mice. In addition, acetylcarnitine was significantly increased in *Wrn* ^*-/-*^ mice compared to WT animals (Fig 3D). The sphingomyelin SM C24:1 was significantly increased in *Wrn* ^*-/-*^ mice compared to *Wrn* ^*Δhel/Δhel*^ mice (Fig 3E) and had a tendency to be higher compared to WT animals. In contrast, the sphingomyelin SM (OH) C22:1 was significantly reduced in the serum of *Wrn* ^*-/-*^ mice compared to both WT and *Wrn* ^*Δhel/Δhel*^ mice (Fig 3F). The other lipids that exhibited at least a significant 1.5-fold difference between our cohorts of mice were classified into two different groups. Table 2 gives a list of these metabolites. One group of metabolites showed increased concentrations in both *Wrn* ^*Δhel/Δhel*^ and *Wrn* ^*-/-*^ mice compared to WT animals (S2 Fig). A second group of metabolites exhibited an increase in serum concentration only in the *Wrn* ^*-/-*^ mice (S3 Fig). Overall, very long chain (> 26 carbons) lysophosphatidylcholines were increased in both *Wrn* ^*Δhel/Δhel*^ and *Wrn* ^*-/-*^ mice compared to WT animals. Shorter chain (< 18 carbons) lysophosphatidylcholines were increased in *Wrn* ^*-/-*^ mice compared to both WT and *Wrn* ^*Δhel/Δhel*^ animals (Table 2). Mainly, saturated and monounsaturated fatty acids were increased in both *Wrn* ^*Δhel/Δhel*^ and *Wrn* ^*-/-*^ mice compared to WT animals. *Wrn* ^*-/-*^ mice showed more polyunsaturated fatty acids with elevated serum concentrations compared to *Wrn* ^*Δhel/Δhel*^ mice (Table 2). One serum prostaglandin (PGE2) was significantly increased in *Wrn* ^*-/-*^ mice compared to both WT and *Wrn* ^*Δhel/Δhel*^ animals (S3 Fig). Thus, the different mutations in the mouse *Wrn* gene lead to different serum lipid profiles.

**Table 2:** Fatty acids, sphingomyelins, and eicosanoids showing significant differences between the different *Wrn* mutants and WT mice.

**Metabolites concentrations higher in both *Wrn*** ^***Δhel/Δhel***^ **and *Wrn*** ^***-/-***^ **compared to WT mice**				
**Metabolites**	**WT levels**	***Wrn*** ^***Δhel/Δhel***^ **levels**	***Wrn*** ^***-/-***^ **levels**	**ANOVA**
lysoPC a C26:0	0.14 ± 0.04	0.41 ± 0.24	0.47 ± 0.18	0.0110
lysoPC a C26:1	0.07 ± 0.02	0.20 ± 0.14	0.22 ± 0.08	0.0304
lysoPC a C28:0	0.18 ± 0.02	0.45 ± 0.16	0.45 ± 0.12	0.0009
lysoPC a C28:1	0.14 ± 0.02	0.42 ± 0.27	0.54 ± 0.22	0.0114
PC ae C30:0	0.16 ± 0.01	0.29 ± 0.10	0.27 ± 0.06	0.0161
PC ae C30:1	0.10 ± 0.03	0.18 ± 0.08	0.20 ± 0.07	0.0298
PC ae C30:2	0.07 ± 0.01	0.12 ± 0.03	0.13 ± 0.02	0.0003
PC ae C36:0	0.44 ± 0.05	0.69 ± 0.23	0.63 ± 0.06	0.0178
PC ae C36:1	2.72 ± 0.33	4.09 ± 1.06	4.72 ± 0.51	0.0007
PC ae C38:1	1.05 ± 0.11	1.76 ± 0.37	2.08 ± 0.35	0.0001
PC aa C28:1	0.13 ± 0.02	0.35 ± 0.13	0.61 ± 0.08	0.0001
**Metabolites concentrations higher in *Wrn*** ^***-/-***^ **mice than in both *Wrn*** ^***Δhel/Δhel***^ **and WT mice**				
**Metabolites**	**WT levels**	***Wrn*** ^***Δhel/Δhel***^ **levels**	***Wrn*** ^***-/-***^ **levels**	**ANOVA**
lysoPC a C16:0	192 ± 28	180 ± 18	272 ± 38	0.0001
lysoPC a C16:1	6.14 ± 1.59	5.25 ± 1.25	9.72 ± 3.12	0.0071
lysoPC a C17:0	2.67 ± 0.49	3.02 ± 0.33	4.07 ± 0.47	0.0001
lysoPC a C18:1	43.7 ± 10.2	37.0 ± 8.3	62.1 ± 12.3	0.0023
lysoPC a C18:2	160 ± 23	148 ± 16	224 ± 25	0.0001
lysoPC a C20:3	10.1 ± 4.3	8.8 ± 3.7	14.9 ± 3.2	0.0358
PC ae C40:5	1.06 ± 0.11	1.13 ± 0.15	1.72 ± 0.07	0.0001
PC ae C44:5	0.18 ± 0.02	0.19 ± 0.04	0.32 ± 0.05	0.0001
PC aa C24:0	0.06 ± 0.01	0.10 ± 0.06	0.14 ± 0.07	0.0462
PC aa C34:2	349 ± 40	366 ± 38	358 ± 58	0.0001
SM C20:2	0.02 ± 0.02	0.03 ± 0.03	0.11 ± 0.04	0.0004
PGE2	44.3 ± 20.0	27.5 ± 20.0	124.2 ± 86.7	0.0142

Values are in μM ± SD except for PGE2 which is in nM.

Since *Wrn* mutant mice may exhibit systemic oxidative stress, we measured the levels of ascorbate and GSH in the blood of these animals. Serum ascorbate was not significantly increased in *Wrn* ^*Δhel/Δhel*^ and *Wrn* ^*-/-*^ mice compared to WT animals (S4A Fig). The levels of serum GSH, in return, were significantly increased in both *Wrn* ^*Δhel/Δhel*^ and *Wrn* ^*-/-*^ mice compared to WT animals (S4B Fig).

### Enzymatic activity of the Wrn^Δhel^ mutant protein and localization in MEFs

**Fig 4:**
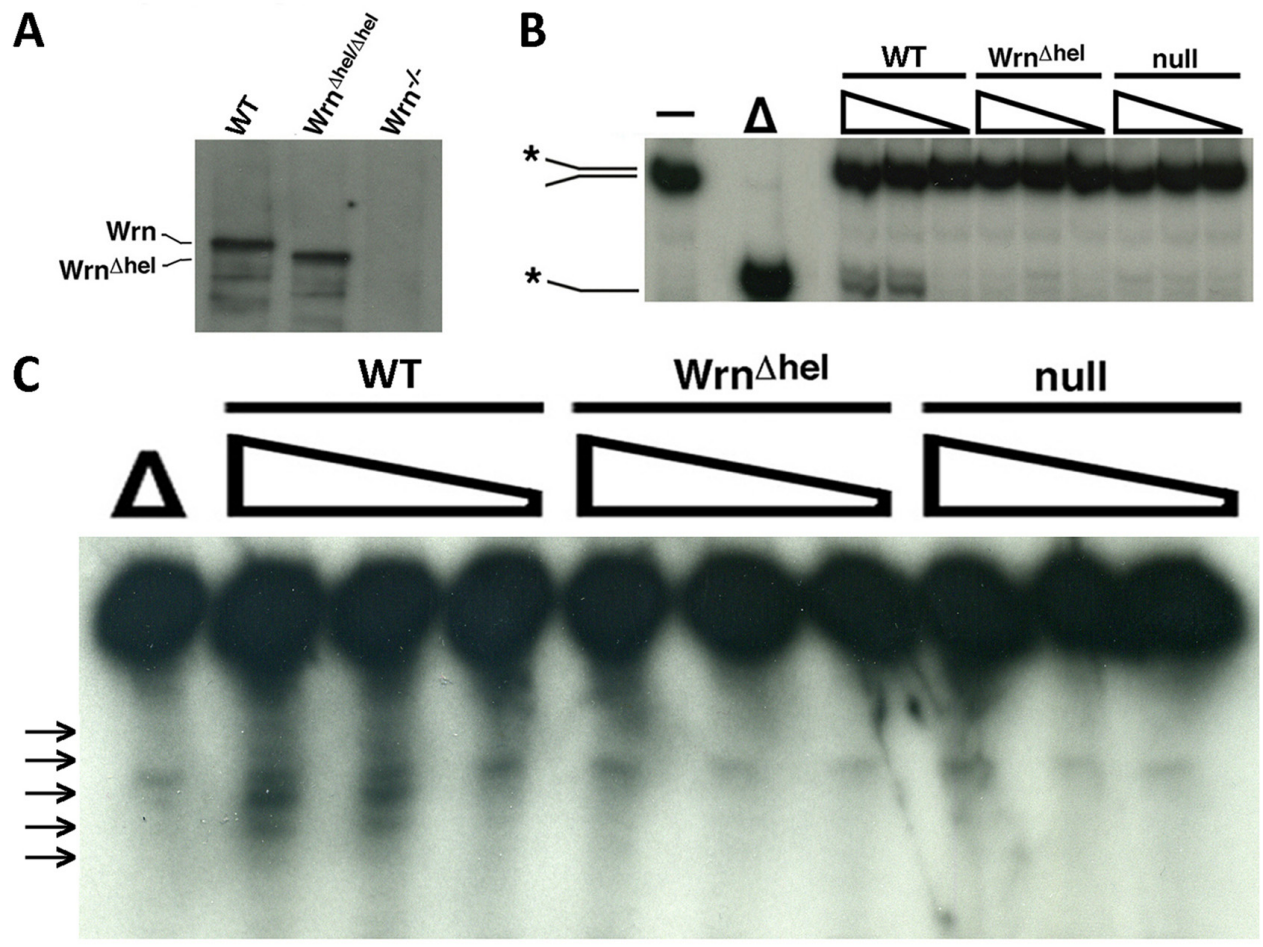
DNA helicase and exonuclease activities of the immunoprecipitated WT and mutant Wrn protein. (A) Example of a western blots is presented. WT, *Wrn* ^*Δhel/Δhel*^ and *Wrn* ^*-/-*^ MEFs were lysed and the Wrn proteins were immunoprecipitated with a mouse anti-Wrn monoclonal antibody. The immunoprecipitates were analyzed by immunoblotting with a rabbit anti-Wrn polyclonal antibody. (B) DNA helicase activity of the immunoprecipitated Wrn proteins. The position of the double strand and single strand radioactive structures are indicated with an asterisk. The “-”lane represents the undenatured double strand radioactive structure. The “Δ” lane represents heat-denatured DNA. (C) Nuclease activity of the immunoprecipitated Wrn proteins. The positions of the cleaved fragments are indicated on the left by arrows. Ten, five, and zero μL of bead solutions containing the immunoprecipitated proteins were used for the enzymatic reactions. The “Δ” lane represents heat-denatured DNA.

To gain clues to how the Wrn^Δhel^ mutant protein would alter several metabolites in mice, we next determined the DNA helicase and exonuclease activities of the Wrn^Δhel^ mutant protein. We first established MEFs from WT, *Wrn* ^*Δhel/Δhel*^, and *Wrn* ^*-/-*^ mice. We then analyzed the Wrn proteins in total cell lysates by immunoprecipitation followed by western blotting. As indicated in Fig 4A, the full-length Wrn protein was immunoprecipitated from WT MEFs. A smaller Wrn^Δhel^ mutant protein was immunoprecipitated from *Wrn* ^*Δhel/Δhel*^ MEFs as described before [14]. No band was detected with the *Wrn* ^*-/-*^ MEFs as previously described for these mutant mice [25]. The immunoprecipitates from the three MEF cultures were incubated with a forked DNA structure. As indicated in Fig 4B, the immunoprecipitated Wrn protein separated the two strands of the DNA substrate. The immunoprecipitated Wrn^Δhel^ mutant protein did not exhibit DNA helicase activity as suspected since the mutation deletes part of the helicase domain [14]. The immunoprecipitates from the *Wrn* ^*-/-*^ MEFs did not show DNA helicase activity in these experiments. The immunoprecipitates from the WT MEFs also displayed a very weak but distinctive nuclease activity. Surprisingly, the immunoprecipitates from the *Wrn* ^*Δhel/Δhel*^ MEFs did not show any exonuclease activity above background level (Fig 4C). This activity was also absent with the immunoprecipitates from the *Wrn* ^*-/-*^ MEFs. These results indicates that the deletion in the Wrn^Δhel^ mutant protein may affect the three dimensional structure of the whole protein and thus the exonuclease catalytic domain of the protein.

**Fig 5:**
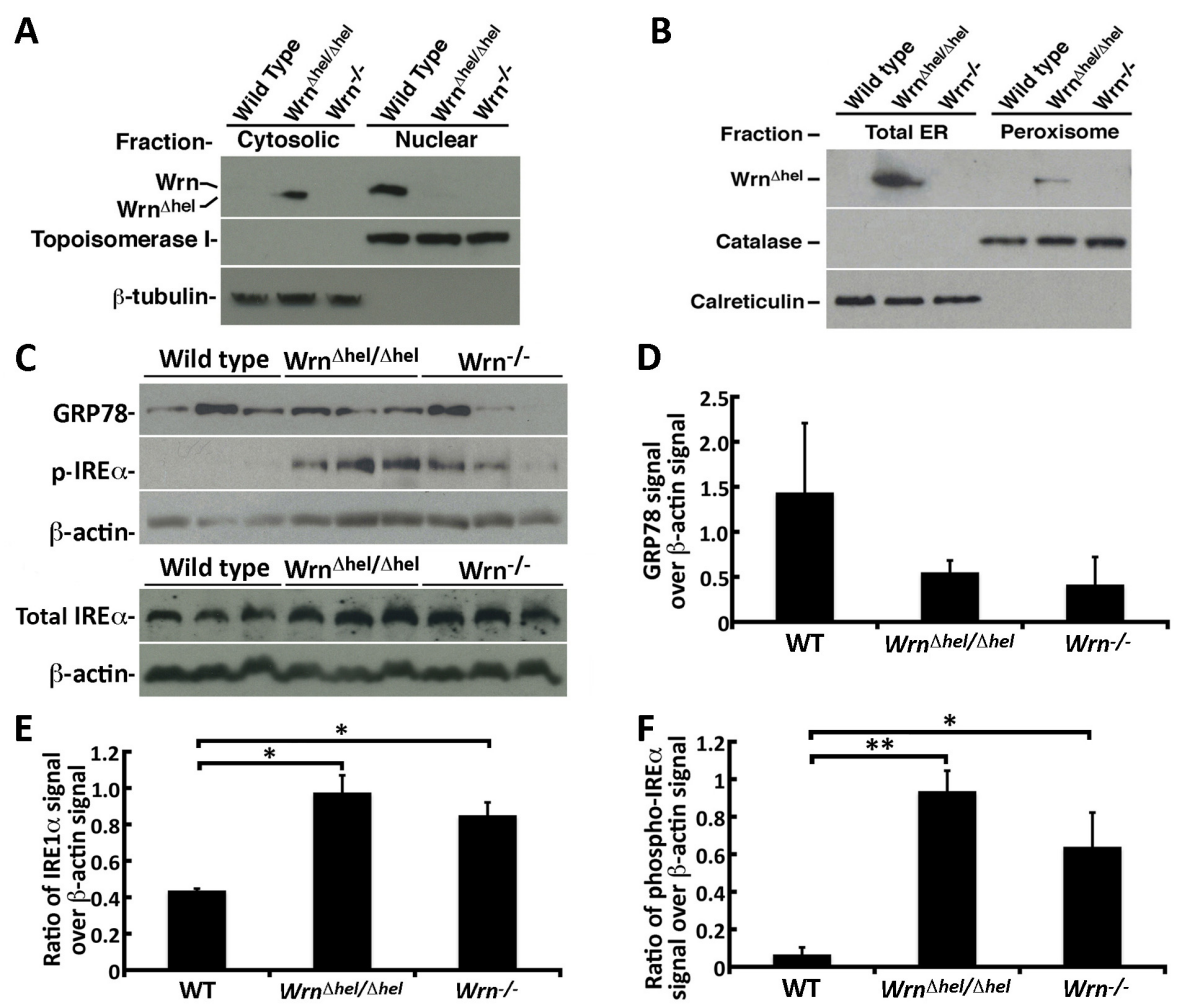
Localization of the WT and mutant Wrn proteins in different subcellular fractions. (A) Example of western blots showing the presence of the Wrn WT protein in the nuclear fraction and the presence of the Wrn^Δhel^ mutant protein mainly in the cytosolic fraction. Fractionations were performed on WT, *Wrn* ^*Δhel/Δhel*^, and *Wrn* ^*-/-*^ MEFs. Topoisomerase I and β-tubulin are nuclear and cytoplasmic markers, respectively. As expected the molecular weight of the Wrn^Δhel^ mutant protein was lower than the Wrn WT protein. No band was detected in *Wrn* ^*-/-*^ MEFs fractions with the anti-Wrn polyclonal antibody. (B) Example of western blots showing only the presence of the Wrn^Δhel^ mutant protein in total ER and the peroxisomal fractions. Fractionations were performed on WT, *Wrn* ^*Δhel/Δhel*^, and *Wrn* ^*-/-*^ liver tissues. Catalase and calreticulin are used as peroxisomal and ER specific markers. This experiment was repeated three times. (C) Protein levels of GRP78, phosphorylated IREα, and β-actin (top panels) and of total IREα, and β-actin (bottom panels) in the spleen of three animals of each genotype. (D) Ratio of GRP78 signal over β-actin signal from western blots. (E) Ratio of IREα over β-actin signal from western blots. (F) Ratio of phosphorylated IREα signal over β-actin signal from western blots. Experiments were performed in triplicates. Bars in all histograms represent SEM. Tukey post ANOVA test *P*-values are shown (*P < 0.05 and **P < 0.01).

Since the three-dimensional structure of the Wrn^Δhel^ mutant protein may be abnormal, we postulated that it might be sorted into different subcellular compartments compared with the normal protein, and so we next determined the localization of this mutant protein in MEFs by cellular fractionation experiments. MEFs were fractionated into nuclear and cytoplasmic fractions. As indicated in Fig 5A, the Wrn WT protein was found in the nuclear fraction but not in the cytoplasmic fraction like the nuclear protein topoisomerase I. In contrast, the Wrn^Δhel^ mutant protein was found in the cytoplasmic fraction of *Wrn* ^*Δhel/Δhel*^ MEFs, like the cytoplasmic β-tubulin protein (Fig 5A). We next determined more precisely the subcellular localization of the Wrn^Δhel^ mutant protein using the liver tissues from *Wrn* ^*Δhel/Δhel*^ mice. Livers were fractionated to obtain total endoplasmic reticulum (ER) and peroxisome fractions, cellular organelles important for lipid metabolism. As indicated in the Fig 5B, only the Wrn^Δhel^ mutant protein could be detected in the total ER and the peroxisome fractions with calreticulin (ER marker) and catalase (peroxisome marker). To determine whether there was an induction of ER stress through activation of the unfolded protein response pathway, we measured the levels of the glucose-related protein-78 (GRP78, also known as BiP) in visceral fat, spleen, heart, kidney, and liver tissues. No significant difference was noted between the different cohorts. A representative western blot showing GRP78 levels in the spleen of mice from each cohort is presented in Fig 5C. A histogram of the signal obtained by western blotting is shown in Fig 5D. We next examined the levels of total IREα and phosphorylated IREα in these tissues, which is another marker of ER stress due to abnormal accumulation of unfolded proteins. The only tissue that showed a significant difference between the genotypes was the spleen (Fig 5C). We could see a significant increase of total IREα in the spleen of *Wrn* ^*Δhel/Δhel*^ and *Wrn* ^*-/-*^ mice compared to WT animals (Fig 5C and 5E). Phosphorylated IREα in the spleen of *Wrn* ^*Δhel/Δhel*^ and *Wrn* ^*-/-*^ mice was also significantly increased compared to WT animals (Fig 5C and 5F). Finally, the levels of phosphorylated IREα in the spleen of *Wrn* ^*Δhel/Δhel*^ mice tended to be higher than in *Wrn* ^*-/-*^ mice (Fig 5F). These results indicate an activation of the unfolded protein response pathway in the spleens of *Wrn* ^*Δhel/Δhel*^ mice.

**Fig 6:**
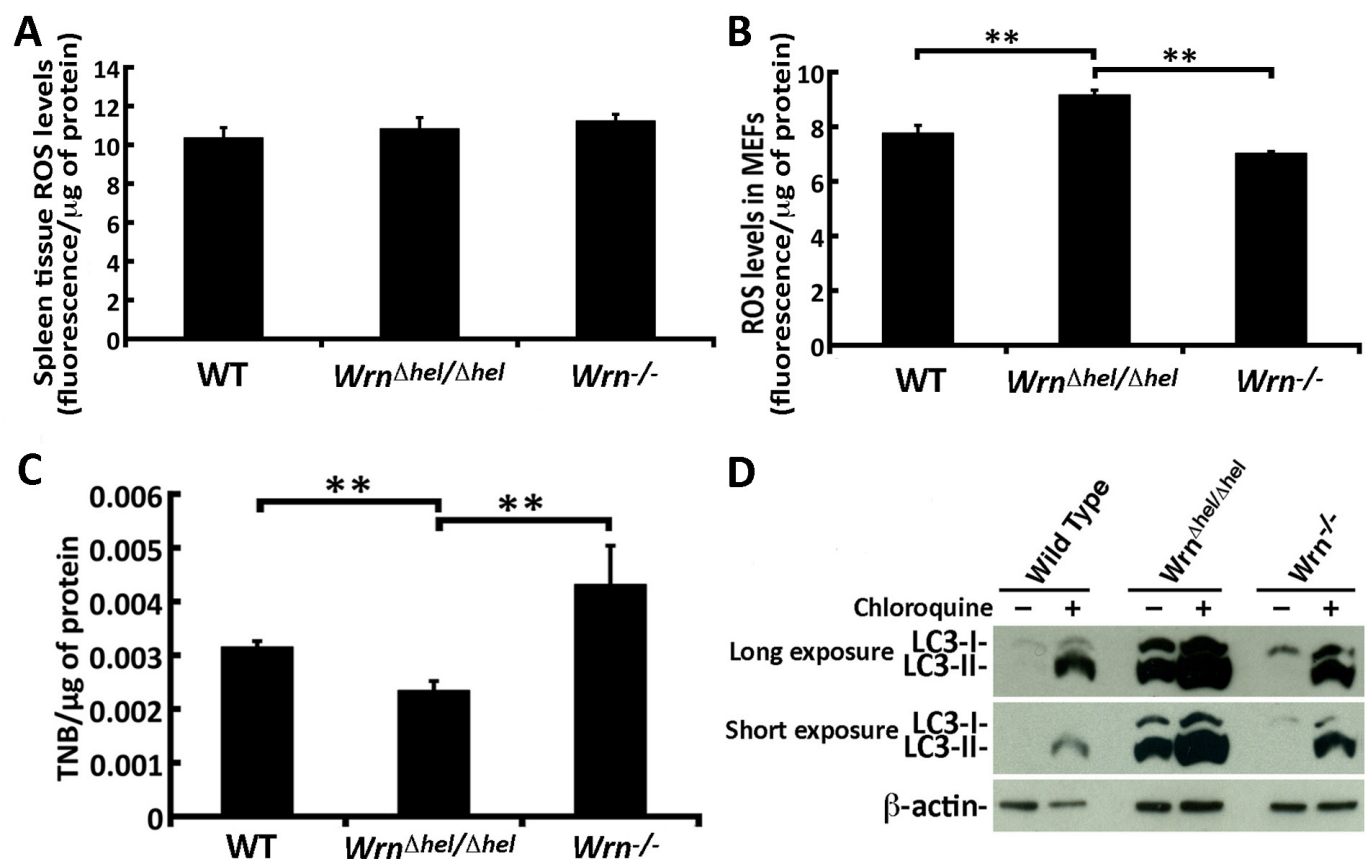
ROS levels, oxidized proteins, and autophagy in WT, *Wrn* ^*Δhel/Δhel*^, and *Wrn* ^*-/-*^ MEFs. (A) ROS levels in spleen tissues of WT, *Wrn* ^*Δhel/Δhel*^, and *Wrn* ^*-/-*^ mice. (B) ROS levels in WT, *Wrn* ^*Δhel/Δhel*^, and *Wrn* ^*-/-*^ MEFs. (C) Quantification of thiol groups on proteins extracted from MEFs by the DTNB assay. Experiments were performed in triplicates. Bars in all histograms represent SEM. Tukey post ANOVA test *P*-values are shown (*P < 0.05 and **P < 0.01). (D) Autophagy flux in WT, *Wrn* ^*Δhel/Δhel*^, and *Wrn* ^*-/-*^ MEFs. Example of western blots showing the increase of LC3-II isoforms in mutant MEFs and in all cells treated with chloroquine (an inhibitor of autophagy flux). All experiments were repeated twice.

As ER stress due to the accumulation of unfolded proteins could impact on the oxidation status, we first measured the levels of ROS in spleen tissues with DCFA. We observed a 10–12% increase in ROS in the spleens of both mutant mice but it was not statistically significant compared to wild type animals (Fig 6A). Since spleen tissues are composed of different cell types, we next decided to measure ROS levels in MEFs. ROS levels were significantly increased in *Wrn* ^*Δhel/Δhel*^ MEFs compared to both WT and *Wrn* ^*-/-*^ MEFs (Fig 6B). It is possible that ROS reacts directly with the cysteine thiols of unfolded proteins to convert them to sulfenic acids [34]. We thus extracted proteins from exponentially growing MEFs and measured the levels of intact thiols on proteins with the DTNB reagent [35]. As indicated in Fig 6C, the levels of intact thiols on proteins were significantly lower in *Wrn* ^*Δhel/Δhel*^ MEFs than in WT or *Wrn* ^*-/-*^ MEFs. These results indicate that the presence of the Wrn^Δhel^ mutant protein in the ER of MEFs affects the levels of intact thiols on proteins in general.

### Abnormal autophagy in Wrn mutant cells

We next examined autophagy by monitoring the microtubule-associated protein light chain 3 (LC3-I and LC3-II) isoforms in exponentially growing MEFs from all three genotypes. The levels and ratios of the isoforms provide information on the flux of autophagy in cells. The amount of LC3-II indicates an increase in autophagosomes in a cell. An increase in the ratio of LC3-II/LC3-I indicates a reduction in the flux of autophagy [36]. As a control experiment, we inhibited the final steps of autophagy with 50 μM of chloroquine. As indicated in Fig 6D, chloroquine caused the accumulation of the LC3-II isoform in all cells thus demonstrating reduced autophagic flux. However, the amount of both isoforms were increased in the *Wrn* ^*Δhel/Δhel*^ MEFs compared to both WT and *Wrn* ^*-/-*^ MEFs (Fig 6D). Different exposures of the western blots showed that autophagy was more affected in *Wrn* ^*Δhel/Δhel*^ MEFs than *Wrn* ^*-/-*^ MEFs. These results indicate that *Wrn* ^*Δhel/Δhel*^ MEFs not only exhibit a significant increase in autophagy but also an aberration in the autophagic flux. Such results were obtained only with MEFs in the exponential phase of the culture. Confluent *Wrn* ^*Δhel/Δhel*^ and *Wrn* ^*-/-*^ MEF cultures did not show significant differences in the levels of LC3 isoforms (S5 Fig).

### Localization of tagged Wrn^Δhel^ mutant protein in MEFs by immunofluorescence study

**Fig 7:**
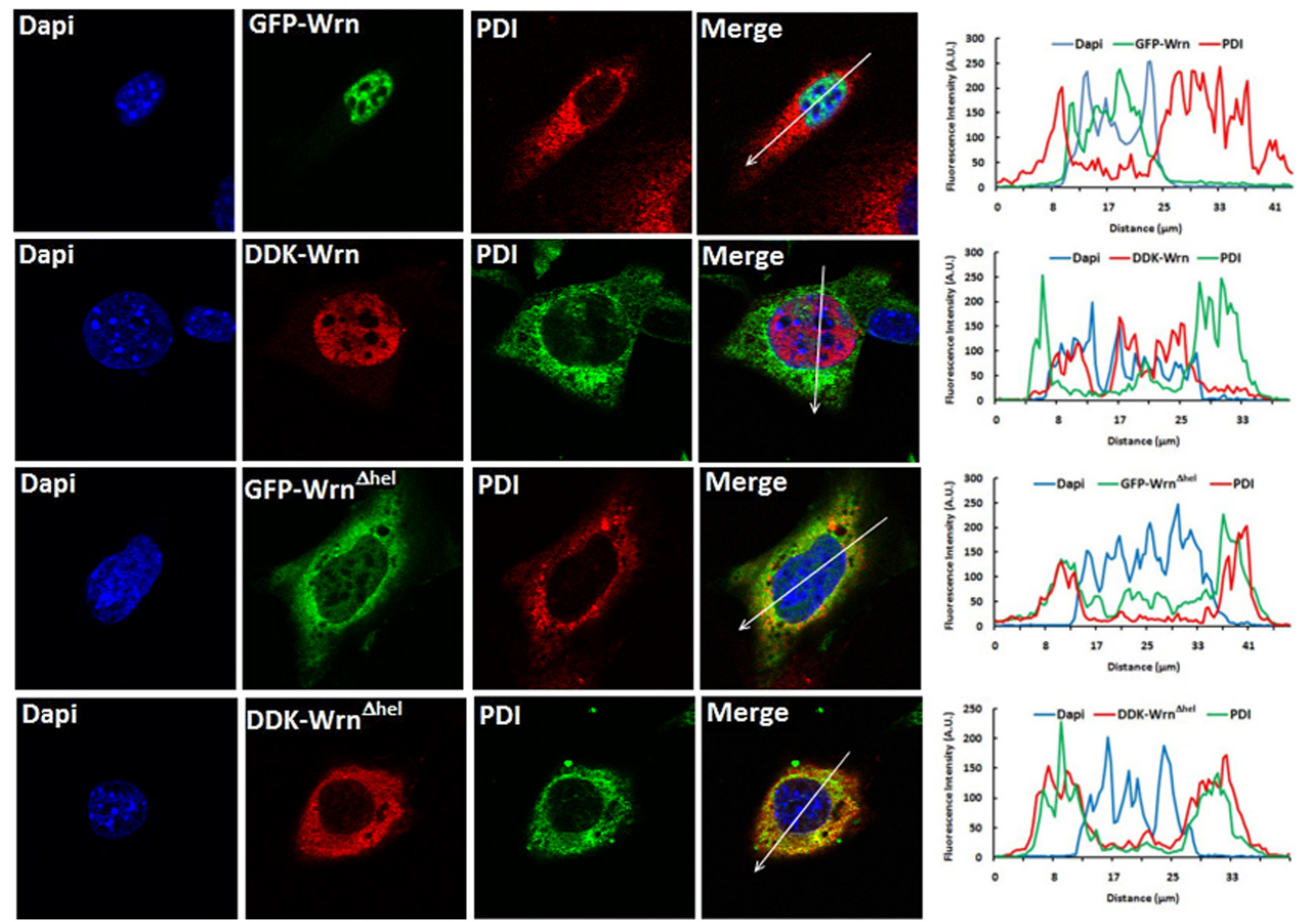
Example of co-localization of the Wrn^Δhel^ mutant protein with the ER organelles by immunofluorescence study. Images in the first row represent the localization of the GFP-Wrn and PDI in *Wrn* ^*Δhel/Δhel*^ MEFs. Images in the second row represent the localization of the DDK-Wrn and PDI in *Wrn* ^*Δhel/Δhel*^ MEFs. Images in the third row represent the localization of the GFP-Wrn^Δhel^ mutant protein and PDI in *Wrn* ^*Δhel/Δhel*^ MEFs. Images in the fourth row represent the localization of the DDK-Wrn^Δhel^ mutant protein and PDI in *Wrn* ^*Δhel/Δhel*^ MEFs. The graph at the end of each row represents the intensity of the fluorescence along the arrow in the merge image.

To complement the subcellular fractionation results, tagged versions of the mouse Wrn and Wrn^Δhel^ mutant constructs were created and transfected into MEFs for immunofluorescence studies with antibodies against protein specific organelles. S6A and S6B Fig show the expression of the Wrn and Wrn^Δhel^ mutant proteins tagged with the GFP at their N-terminus or the hemagglutinin DDK sequence at their C-terminus end. Full-length protein constructs were detected by western blot analyses with both tags. As indicated in Fig 7, the WT GFP-Wrn and DDK-Wrn proteins were exclusively nuclear and did not co-localized with the ER marker PDI protein. The nuclear distribution of the Wrn WT protein was the same with both tags (GFP or DDK) and in both *Wrn* ^*Δhel/Δhel*^ (Fig 7) and WT MEFs (S7 Fig). In contrast, the GFP-Wrn^Δhel^ mutant protein mainly localized with the PDI marker in the cytoplasm. Similar results were obtained with the DDK-Wrn^Δhel^ mutant protein. The merged images and the graphs showed good co-localization between the tagged Wrn^Δhel^ mutant protein and PDI. S8 Fig shows examples of localizations of the tagged Wrn proteins with the peroxisomal (catalase), lysosomal (Lamp1), and mitochondrial (AIF) markers. Table 3 indicates that the highest co-localization (based on the calculated Pearson’s correlation coefficients) is with the ER marker for both tagged forms of the Wrn^Δhel^ mutant protein. Table 2 also shows a significant co-localization with the peroxisomal marker catalase.

**Table 3:** Pearson's correlation coefficients between the tagged Wrn proteins and the indicated organelle markers^a^.

	GFP-Wrn	GFP-Wrn^Δhel^	DDK-Wrn	DDK-Wrn^Δhel^
ER (PDI)	0.046 ± 0.065	0.741 ± 0.031^b^	0.097 ± 0.058	0.814 ± 0.017^b^
Peroxisome (Catalase)	0.473 ± 0.043	0.644 ± 0.037^c^	0.242 ± 0.054	0.675 ± 0.029^c^
Lysosome (Lamp-1)	0.237 ± 0.089	0.562 ± 0.037	0.242 ± 0.054	0.564 ± 0.055
Mitochondria (AIF)	0.046 ± 0.037	0.416 ± 0.036	-0.030 ± 0.042	0.612 ± 0.024

^a^ Means of the calculated Pearson’s are indicated with the SEM. N = 3–5 cells for the GFP-Wrn and the DDK-Wrn constructs. N = 5–7 cells for the GFP-Wrn^Δhel^ and the DDK-Wrn^Δhel^ constructs.

^b^ *P* < 0.05.

^c^ *P* < 0.1.

## Discussion

It has been reported that mice unable to accumulate any Wrn protein (*Wrn* null mice) do not exhibit a reduction in their life span compared to WT animals [25]. In contrast, mice synthesizing a stable Wrn protein lacking part of its helicase domain show a reduction in their mean life span compared to WT animals [18]. In this study, we further characterized the difference between these *Wrn* mutant mice at the cellular and molecular level. Survival curves re-confirmed the shorter mean life span of *Wrn* ^*Δhel/Δhel*^ mice compared not only to WT animals but also to *Wrn* ^*-/-*^ mice (Fig 1A). The mean and maximum life span of *Wrn* ^*-/-*^ and WT mice were similar. Surprisingly, we found that *Wrn* ^*-/-*^ males were on average 5–13% bigger than both WT and *Wrn* ^*Δhel/Δhel*^ mice during the first year of their life. There was an increase in visceral adipose tissues in *Wrn* ^*-/-*^ males compared to both *Wrn* ^*Δhel/Δhel*^ and WT males but this was not statistically significant. We noticed a significant increase at four months of age in the wet weight of the spleen in both types of *Wrn* mutant mice compared to age-matched WT mice. These results may suggest an increased pro-inflammatory status in both *Wrn* helicase and null mutant mice. Perhaps related, compared with WT animals, we found a significant increase in blood lymphocyte counts in *Wrn* ^*-/-*^ mice and a similar, though non-significant, trend in *Wrn* ^*Δhel/Δhel*^ mice. It has been reported that rodents exhibiting dyslipidemia show an increase in spleen weight, which is consistent with our observation [37]. Chronic dyslipidemia is known to lead to systemic low-grade inflammation [38].

Intriguingly, both *Wrn* ^*Δhel/Δhel*^ and *Wrn* ^*-/-*^ mice display a low platelet count. Based on the platelet counts, all *Wrn* mutant mice exhibit a moderate thrombocytopenia that may not be life threatening, as *Wrn* ^*-/-*^ mice did not have a reduced life span compared to WT mice. In addition, red blood cell count and the % hematocrit were ~20% lower in *Wrn* ^*Δhel/Δhel*^, but not *Wrn* ^*-/-*^, mice compared to WT animals. Thus, the larger spleen in *Wrn* ^*Δhel/Δhel*^ mice compared to both WT and *Wrn* ^*-/-*^ mice correlated with a low but chronic state of anemia in *Wrn* ^*Δhel/Δhel*^ mice. There is no evidence for kidney disease causing the anemia. In addition, the levels of serum erythropoietin were not lower in the two different *Wrn* mutant types compared to WT cohort. Therefore, splenic sequestration or destruction of red blood cells in *Wrn* ^*Δhel/Δhel*^ mice may underlie our findings.

*Wrn* ^*-/-*^ mice showed a significant decrease in serum MIP-1α (macrophage inflammatory protein-1α) compared to WT animals, and *Wrn* ^*Δhel/Δhel*^ mice displayed a similar trend. MIP-1α acts as a chemoattractant to a variety of cells including monocytes, T cells, B cells, and eosinophils. In addition, it inhibits the proliferation of hematopoietic stem cells *in vitro* and *in vivo* [39]. In contrast, IL-18 is significantly decreased in the serum of *Wrn* ^*Δhel/Δhel*^ mice compared to WT animals, whereas *Wrn* ^*-/-*^ mice showed a similar trend. A lower secretion of IL-18 has been shown to be an important factor in the dysfunction of dendritic cells in aged C57BL/6 mice [40]. Dendritic cells are central in regulating both innate and acquired immunity. A low secretion of IL-18 can lead to the failure of natural killer cell activation and of tumor eradication in old mice [40]. IL-10 is significantly increased in the serum of *Wrn* ^*Δhel/Δhel*^ mice compared to both WT and *Wrn* ^*-/-*^ mice. IL-10 is the only anti-inflammatory cytokine that showed a major difference between *Wrn* ^*Δhel/Δhel*^ and *Wrn* ^*-/-*^ mice. IL-10 is a negative regulation of myeloid dendritic cell activation [41] and a negative regulation of B-cell proliferation [42]. Finally, it has been reported that the majority of IL-10 secreted in the serum is synthesized by the spleen in rodents [38] and acts as an anti-inflammatory cytokine modulating other cytokines during the inflammation of different age-related chronic diseases [38, 43, 44]. Without IL-10 responses (such as in IL-10 knock out mice), the levels of pro-inflammatory cytokines increase [38, 43, 44]. Notably, one study indicated that in a mouse model of chronic inflammatory liver disease serum IL-10 levels correlated with the size of the spleen during the course of the disease [45]. A longitudinal study with animals of different ages will be required to follow the evolution of the inflammatory cytokine levels in different organs in these mutant mice compared to wild type animals. Noticeably, we previously found a significant increase in the expression of a set of genes involved in inflammatory response in older nine month-old *Wrn* ^*Δhel/Δhel*^ mice [18]. Of important relevance, analysis of the human C-reactive protein has revealed a minor but chronic inflammation-driven aging mechanism in WS patients [46].

The plasminogen activator inhibitor-1 (PAI-1) is only significantly increased in the serum of *Wrn* ^*Δhel/Δhel*^ mice. PAI-1 is a serine protease inhibitor that functions as the principal inhibitor of tissue plasminogen activator and urokinase, the activators of plasminogen and hence the physiological breakdown of blood clots [47]. In inflammatory conditions, PAI-1 is secreted by endothelial cells and appears to play a significant role in the progression to fibrosis [48, 49]. Importantly, PAI-1 is present in increased levels in various disease states such as cardiovascular diseases (during the development of vessel wall damage) [50], metabolic syndrome [51], and remarkably in human Werner syndrome [52]. It is considered a marker of inflammation by some authors [51]. Furthermore, an increase in serum PAI-1 is associated with aging in several animal models [53], in the aging human population [54], and it is also elevated in senescent cells [55, 56]. Finally, it is significantly upregulated in a variety of pathologies associated with the process of aging, including myocardial and cerebral infarction, atherosclerosis, cardiac and lung fibrosis, metabolic syndromes (e.g., hypertension, hyperlipidemia, and insulin resistance), cancer, and inflammatory/stress responses [54]. Thus, the over secretion of serum PAI-1 may disadvantage *Wrn* ^*Δhel/Δhel*^ mice compared to *Wrn* ^*-/-*^ mice. Noticeably, cardiovascular fibrosis has been reported in aged *Wrn* ^*Δhel/Δhel*^ mice [24]. Although more thorough analyses are required, we surveyed a small group of *Wrn* ^*-/-*^ mice and did not detect cardiovascular anomalies. The platelet compartment is a major site of PAI-1 synthesis in the blood [57]. Since we detected a decrease in platelet counts in Wrn mutant mice, another likely site of PAI-1 synthesis is the endothelial compartment. The molecular reason for increased PAI-1 secretion in *Wrn* ^*Δhel/Δhel*^ mice is unknown at present, but may be due to the cellular stress caused by a mislocalization of the mutant Wrn^Δhel^ protein in the endothelial cells of *Wrn* ^*Δhel/Δhel*^ mice (see below).

As we previously observed metabolic anomalies in aging *Wrn* ^*Δhel/Δhel*^ mice [16, 18], in this study we compared the metabolomic profiles of these mutants with both WT and *Wrn* ^*-/-*^ mice. Three metabolites were significantly altered in *Wrn* ^*Δhel/Δhel*^ mice compared to both WT and *Wrn* ^*-/-*^ mice. Serum hydroxyproline (OH-Pro) and the phosphatidylcholine PC aa C30:2 were significantly increased in *Wrn* ^*Δhel/Δhel*^ mice compared to both WT and *Wrn* ^*-/-*^ mice. OH-Pro is a major component of the protein collagen and plays key role in collagen stability. A variety of disease states are believed to affect collagen turnover and can cause elevated serum or urine OH-Pro [58]. In addition, chronic inflammation of the liver has been shown to increase OH-Pro [59, 60]. The impact of the abnormal increase in serum phosphatidylcholine PC aa C30:2 on the long-term health of mice is unknown but we observed it only in *Wrn* ^*Δhel/Δhel*^ mice. Interestingly, elevated serum PC aa C30:2 has been associated with severe fatty liver diseases in dairy cows [61]. Thus, the increase in PC aa C30:2 levels in *Wrn* ^*Δhel/Δhel*^ mice maybe a marker precursor of hepatic lipidosis observed in older *Wrn* ^*Δhel/Δhel*^ mice [23]. Finally, serum acetylcarnitine is significantly decreased in *Wrn* ^*Δhel/Δhel*^ mice compared to both WT and *Wrn* ^*-/-*^ mice. Acetylcarnitine is an acetic acid ester of carnitine that facilitates movement of acetyl CoA into the matrices of mammalian mitochondria during the oxidation of fatty acids [62]. Acetylcarnitine also decreases glucose consumption in favor of fat oxidation in non-diabetics [63]. Finally, acetylcarnitine may have antioxidative properties, protecting cells against lipid peroxidation and membrane breakdown [62]. Accordingly, we observed increased lipid peroxidation in aging *Wrn* ^*Δhel/Δhel*^ mice compared to age-matched WT animals [23]. These considerations suggest that *Wrn* ^*Δhel/Δhel*^ mice are at a disadvantage with low levels of acetylcarnitine.

Major differences were found between *Wrn* ^*Δhel/Δhel*^ and *Wrn* ^*-/-*^ mice with regards to serum lipid species. Interestingly, only the very long chain lysophosphatidylcholines (26 and 28 carbons) are abnormally increased in the serum *Wrn* ^*Δhel/Δhel*^ mice compared to WT animals. In contrast, *Wrn* ^*-/-*^ mice exhibit not only an increase in very long chain lysophosphatidylcholines but also in shorter chain lysophosphatidylcholines. Thus, the ratio of very long chains over the short chains lysophosphatidylcholines is abnormally increased in *Wrn* ^*Δhel/Δhel*^ mice compared to *Wrn* ^*-/-*^ mice. Such an observation indicates a perturbation in the β-oxidation of long fatty acids in the peroxisomes [64] of *Wrn* ^*Δhel/Δhel*^ mice. Very long fatty acids (> 22 carbons) are not processed by mitochondria as they are not transported into them for further oxidation [65]. Moreover, mainly saturated and monounsaturated phosphatidylcholine types are increased in *Wrn* ^*Δhel/Δhel*^ mice. In addition to saturated and monounsaturated phosphatidylcholines, *Wrn* ^*-/-*^ mice displayed an increase in several polyunsaturated phsophatidylcholines (Table 1). Thus, although the body weight of *Wrn* ^*-/-*^ mice is 5–13% greater than *Wrn* ^*Δhel/Δhel*^ mice, the number of increased polyunsaturated fatty acid species is close to the number of increased mono and unsaturated fatty acids. In contrast, this balance is perturbed in the serum of *Wrn* ^*Δhel/Δhel*^ mice. Polyunsaturated fatty acids are essential and they reduce risk of heart disease [66]. It is also known that a shift from unsaturated towards saturated fatty acyl chains of membrane phospholipids directly induces a decrease in glucose effectiveness and insulin sensitivity [67]. The lipid disturbance observed in *Wrn* ^*Δhel/Δhel*^ mice may explain in part the abnormal ATP levels, the insulin resistance, the cardiovascular phenotypes eventually seen in older mutant mice, and the shorter life span observed in *Wrn* ^*Δhel/Δhel*^ mice compared to WT and *Wrn* ^*-/-*^ mice.

The metabolic profile in the *Wrn* ^*Δhel/Δhel*^ mice gave a first clue of the impact of a stable Wrn^Δhel^ mutant protein on the observed cellular and molecular anomalies. The deletion that was generated not only affected the Wrn helicase activity but also the exonuclease activity as well as the localization of the mutant protein. These results indicate that the 21 amino acid deletion in the helicase domain of the Wrn protein affected the overall three-dimensional structure of the protein (including the exonuclease domain and the nuclear localization signal). Fractionation and immunofluoresence studies indicated that the Wrn^Δhel^ mutant protein was present in the ER and the peroxisome fraction. The Wrn protein does not contain a peroxisomal localization signal. However, a significant fraction of peroxisomes originates from an ER membrane template [68]. It is thus possible that Wrn^Δhel^ mutant proteins associated with the ER may end up in the peroxisomes and affect the normal function of this organelle, as seen by the abnormal increase in very long chain lysophosphatidylcholines in *Wrn* ^*Δhel/Δhel*^ mice. In addition, the mislocalization of the Wrn^Δhel^ mutant protein also affects the flux of autophagy. Interestingly, *Wrn* ^*-/-*^ MEFs also exhibited a defect in the flux of autophagy as it has been described for human fibroblasts derived from WS patients or from WRN-depleted human cells [69–71]. However, this defect was more severe in *Wrn* ^*Δhel/Δhel*^ MEFs. Importantly, autophagy is involved in the activation of hepatic stellate cells, liver fibrosis, and extracellular matrix modifications [72]. Abnormal autophagy in the endothelial cells of *Wrn* ^*Δhel/Δhel*^ mice is likely a contributor to the abnormal increase in PAI-1 and OH-Pro serum levels, an issue that will need to be explored in the future. The increased levels of OH-Pro also indicate alterations in collagen processing. Disturbance in the normal function of the ER is known to affect collagen processing and secretion into the extracellular matrix [34]. Moreover, the level of intact thiols on proteins is decreased in *Wrn* ^*Δhel/Δhel*^ MEFs indicating that a mislocalization of the Wrn^Δhel^ mutant protein in the ER potentially lead to misoxidized proteins [34] that could accumulate in autophagosomes. It is interesting to note that the defect in autophagy was revealed in actively growing Wrn mutant MEFs in culture but not with confluent cells. This may be explained by the increase demand on the requirement for recycling different cellular components by autophagy in actively growing cells.

## Conclusions

To conclude, we found that the Wrn^Δhel^ mutant protein is mislocalized in cells of *Wrn* ^*Δhel/Δhel*^ mice. This mislocalization affects the normal function of several organelles, including the peroxisomes, the ER, and the autophagosomes, leading potentially to the alteration of serum metabolites. Such alterations may be the precursors of molecular events that are responsible for the age-related changes observed in older *Wrn* ^*Δhel/Δhel*^ mice. One study previously reported that fibroblasts derived from adult transgenic mice expressing a dominant-negative human WRN helicase protein exhibited hypersensitivity to the chemical 4-nitroquinoline-1-oxide and reduced replicative potential *in vitro* [73]. However, no additional information on the phenotype of these transgenic mice was published. This work is the first study comparing *Wrn* ^*Δhel/Δhel*^ and *Wrn* ^*-/-*^ mice side by side at the cellular and tissue levels suggesting that the Wrn^Δhel^ mutant protein may be considered a poison protein with more deleterious effects in the long term than the absence of Wrn protein in null mice. Interestingly, a recent report indicated that WS patients with a nonsense mutation at position 1256 of the human WRN protein synthesized a stable truncated protein localized in the cytoplasm of their cells [74]. These patients exhibited type 2 diabetes, cataracts, hypercholesterolemia, short stature, bird-like facies, skin ulcers on the lower limbs, osteoporosis, and arterial atherosclerosis. A survey of different WS derived cells with different mutations [13] will be required with the appropriate antibodies to assess the impact of abnormal WRN protein in such cells. It will be important to determine whether the phenotype of human WS cells expressing a detectable mislocalized truncated WRN protein is more severe than WS cells with no measurable level of the WRN protein. Of relevance to this work, a clinical study reported that different mutations in the WRN protein were associated with different subtypes of thyroid carcinomas in Japenese patients with WS [75]. Altogether, these results suggest that different mutated WRN protein may in part explain the phenotypic differences between several WS patients.

## Supporting Information

S1 Fig Weight of different organs and levels of serum erythropoietin in WT, *Wrn* ^*Δhel/Δhel*^, and *Wrn* ^*-/-*^ mice. (A) Histogram presenting the total body weight gain during the first year of life (n = 6–9 males). One-way ANOVA: WT *vs*. *Wrn* ^*Δhel/Δhel*^ mice, *P* = 0.175; WT *vs*. *Wrn* ^*-/-*^ mice, *P = 0.035; *Wrn* ^*Δhel/Δhel*^ *vs*. *Wrn* ^*-/-*^ mice, **P = 0.007. (B) Histogram presenting food intake at four months of age. (C) Histogram presenting water consumption at four months of age. (D) Histogram representing the ratio of visceral fat wet weight over total body weight. (E) Histogram representing the ratio of heart wet weight over total body weight. (F) Histogram representing the ratio of liver wet weight over total body weight. (G) Histogram representing the ratio of lung wet weight over total body weight. (H) Histogram representing the ratio of kidney wet weight over total body weight. (I) Histogram representing the levels of serum erythropoietin. Bars in all histograms represent SEM. N = 6 males for each cohort.
(JPG)

S2 Fig Serum levels of eleven serum metabolites significantly increased in *Wrn* ^*Δhel/Δhel*^ and *Wrn* ^*-/-*^ mice compared to WT animals. Bars in all histograms represent SD. N = 6 males for each cohort.
(JPG)

S3 Fig Serum levels of twelve serum metabolites significantly increased in *Wrn* ^*-/-*^ mice compared to WT and *Wrn* ^*Δhel/Δhel*^ mice. Bars in all histograms represent SD. N = 6 males for each cohort.
(JPG)

S4 Fig Serum ascorbate and GSH levels in WT, *Wrn* ^*Δhel/Δhel*^, and *Wrn* ^*-/-*^ mice. Bars in all histograms represent SEM. N = 5 males for each cohort. Tukey post ANOVA test *P*-values are shown (**P < 0.01).
(JPG)

S5 Fig Levels of LC3 isoforms in WT, *Wrn* ^*Δhel/Δhel*^, and *Wrn* ^*-/-*^ MEFs at confluence. The histogram on the right represents the ratio of LC3-II signal over β-tubulin signal from two independent experiments.
(JPG)

S6 Fig Expression of tagged Wrn protein constructs in transfected WT MEFs. (A) Example of a western blot showing the expression of the WT GFP-Wrn and the mutant GFP-Wrn^Δhel^ proteins in total cell lysates. (B) Example of a western blot showing the expression of the immunoprecipitated WT DDK-Wrn and the immunoprecipitated mutant DDK-Wrn^Δhel^ proteins. Proteins were immunoprecipitated with the anti-DDK antibody and revealed by western with an antibody against the Wrn protein.
(JPG)

S7 Fig Example of WT GFP-Wrn localization in WT MEFs. The actin network is revealed by using a fluorescent phalloidin reagent.
(JPG)

S8 Fig Example of co-localization of the Wrn^Δhel^ mutant protein with the ER organelles by immunofluorescence study. Images in the first row represent the localization of the GFP-Wrn^Δhel^ mutant protein and catalase (peroxisomal marker) in *Wrn* ^*Δhel/Δhel*^ MEFs. Images in the second row represent the localization of the DDK-Wrn^Δhel^ protein and catalase in *Wrn* ^*Δhel/Δhel*^ MEFs. Images in the third row represent the localization of the GFP-Wrn^Δhel^ protein and Lamp-1 (lysosomal marker) in *Wrn* ^*Δhel/Δhel*^ MEFs. Images in the fourth row represent the localization of the DDK-Wrn^Δhel^ mutant protein and Lamp-1 in *Wrn* ^*Δhel/Δhel*^ MEFs. Images in the fifth row represent the localization of the GFP-Wrn^Δhel^ mutant protein and AIF (mitochondrial marker) in *Wrn* ^*Δhel/Δhel*^ MEFs. Images in the sixth row represent the localization of the DDK-Wrn^Δhel^ mutant protein and AIF in *Wrn* ^*Δhel/Δhel*^ MEFs. The graph at the end of each row represents the intensity of the fluorescence along the arrow in the merge image.
(JPG)

S1 Table Metabolic names and codes. (XLSX)

S2 Table Serum levels of cytokines, metabolic hormones, and cardiovascular factors. (XLSX)

S3 Table Serum levels of metabolites. Metabolites include amino acids and biogenic amines, acylcarnitines, lysophosphatidylcholines, phophatidylcholines, sphingomyelins, hexoses, and prostaglandins.
(XLSX)
