# Supplementary figures and images for: Metabolic and Phenotypic Differences between Mice Producing a Werner Syndrome Helicase Mutant Protein and Wrn Null Mice
Source: PLoS One. 2015 Oct 8;10(10):e0140292. doi: 10.1371/journal.pone.0140292 (PMC4598085; doi:10.1371/journal.pone.0140292)

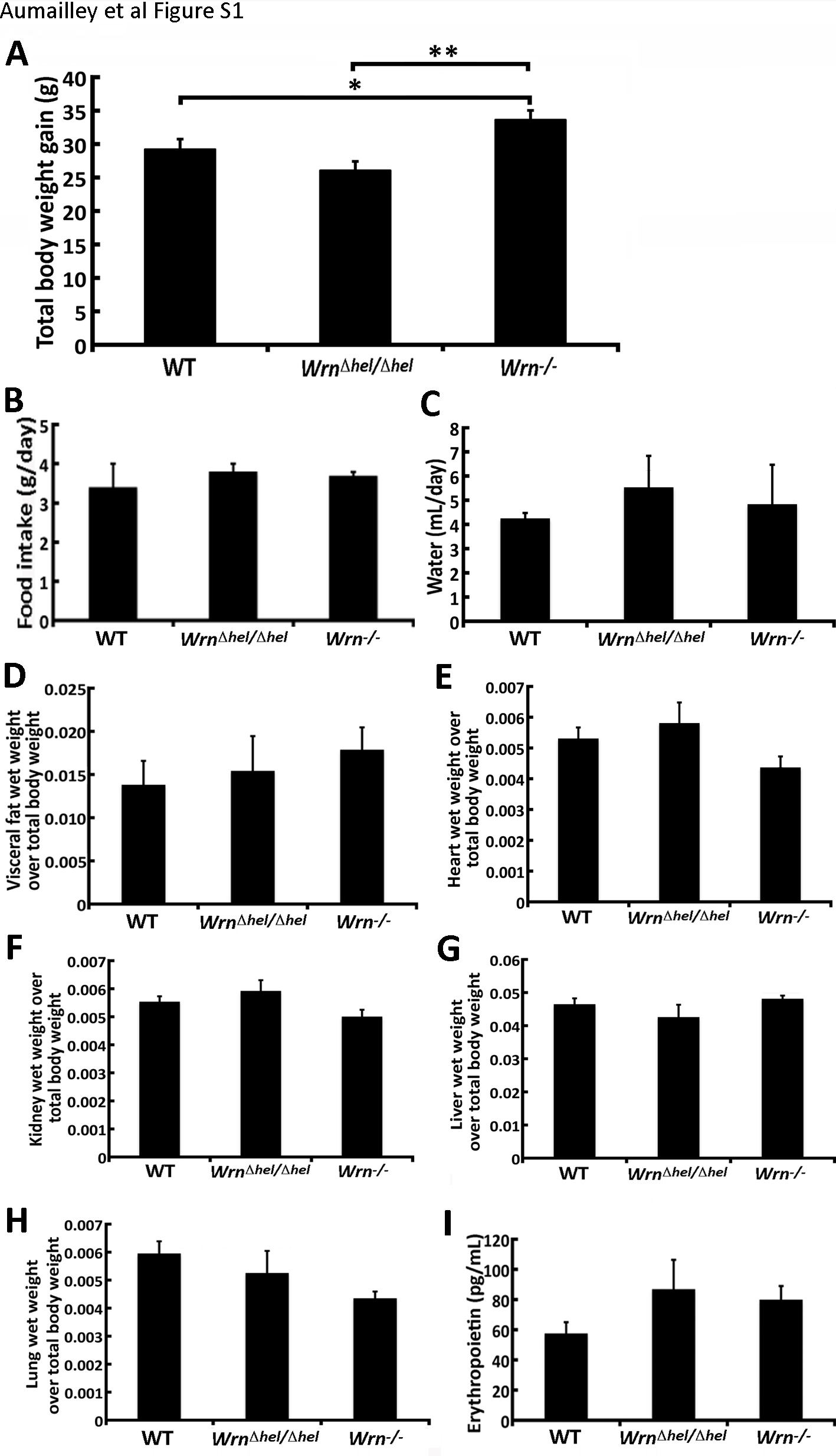

Supplement: S1 Fig — (A) Histogram presenting the total body weight gain during the first year of life (n = 6–9 males). One-way ANOVA: WT vs. Wrn Δhel/Δhel mice, P = 0.175; WT vs. Wrn -/- mice, *P = 0.035; Wrn Δhel/Δhel vs. Wrn -/- mice, **P = 0.007. (B) Histogram presenting food intake at four months of age. (C) Histogram presenting water consumption at four months of age. (D) Histogram representing the ratio of visceral fat wet weight over total body weight. (E) Histogram representing the ratio of heart wet weight over total body weight. (F) Histogram representing the ratio of liver wet weight over total body weight. (G) Histogram representing the ratio of lung wet weight over total body weight. (H) Histogram representing the ratio of kidney wet weight over total body weight. (I) Histogram representing the levels of serum erythropoietin. Bars in all histograms represent SEM. N = 6 males for each cohort. (JPG) [file pone.0140292.s001.jpg]

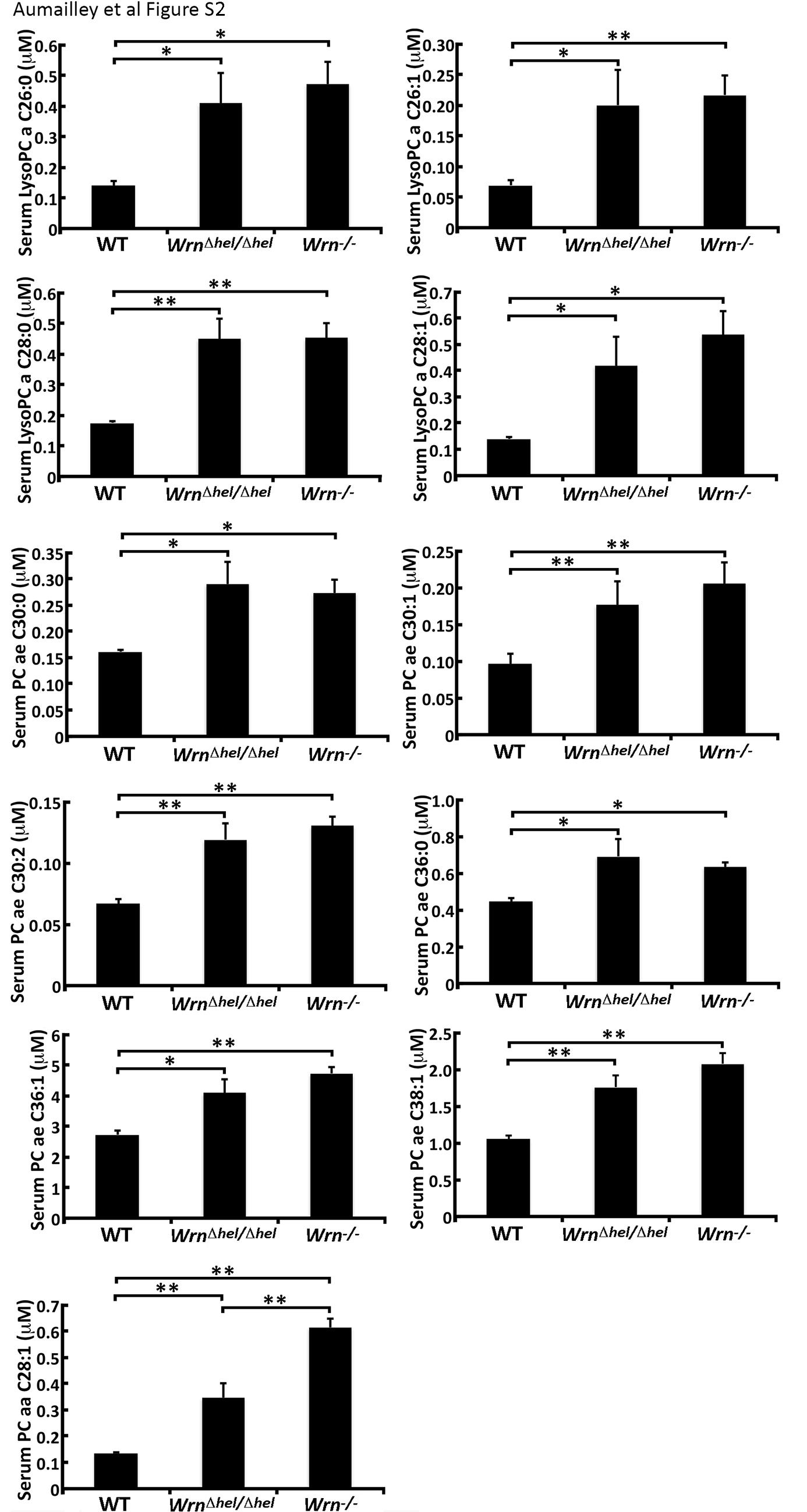

Supplement: S2 Fig — Bars in all histograms represent SD. N = 6 males for each cohort. (JPG) [file pone.0140292.s002.jpg]

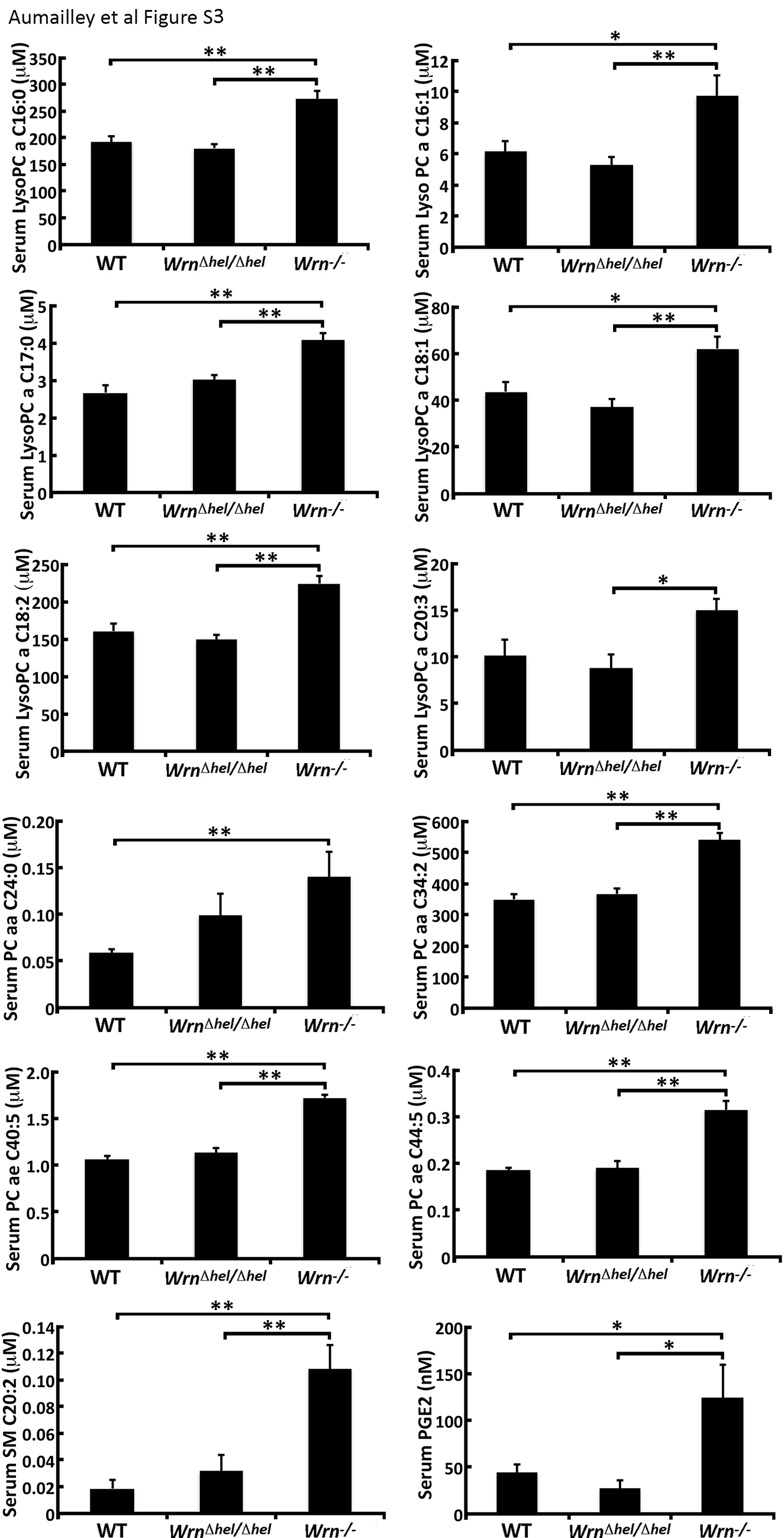

Supplement: S3 Fig — Bars in all histograms represent SD. N = 6 males for each cohort. (JPG) [file pone.0140292.s003.jpg]

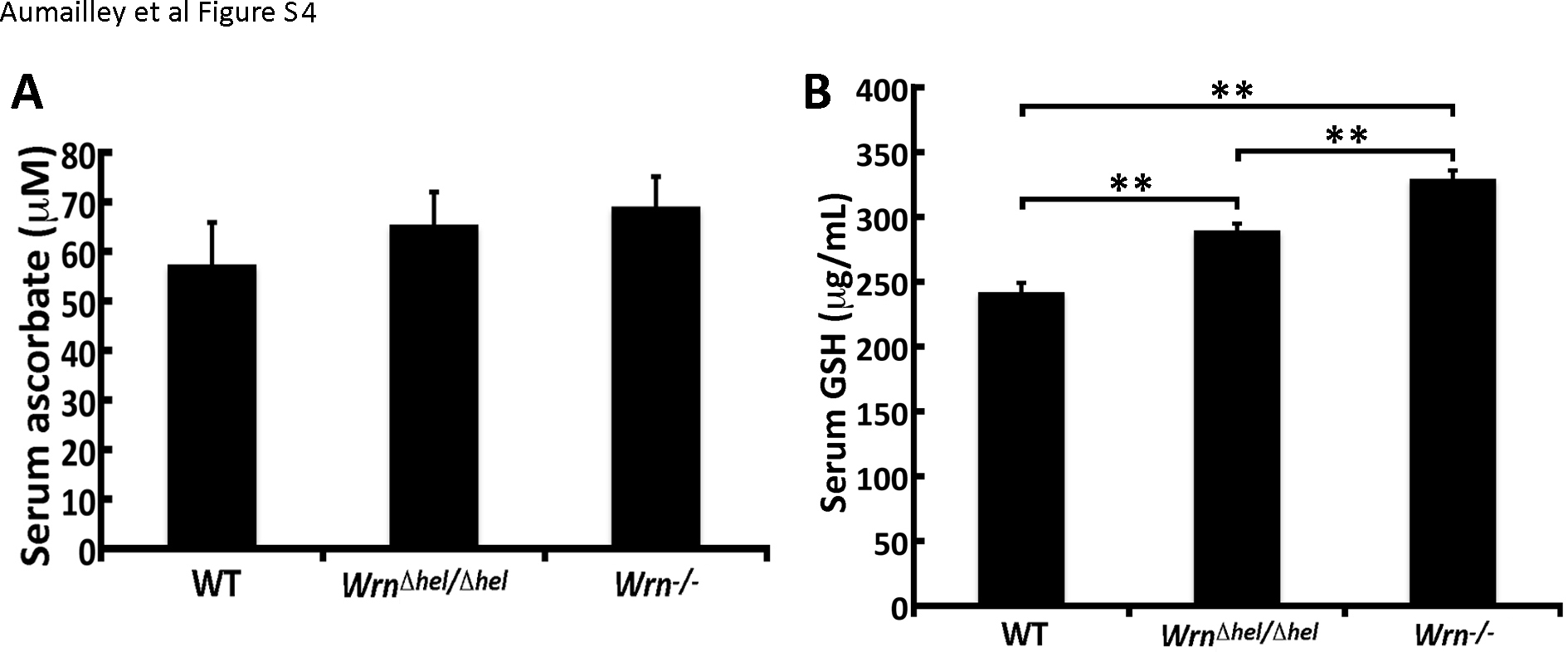

Supplement: S4 Fig — Bars in all histograms represent SEM. N = 5 males for each cohort. Tukey post ANOVA test P-values are shown (**P < 0.01). (JPG) [file pone.0140292.s004.jpg]

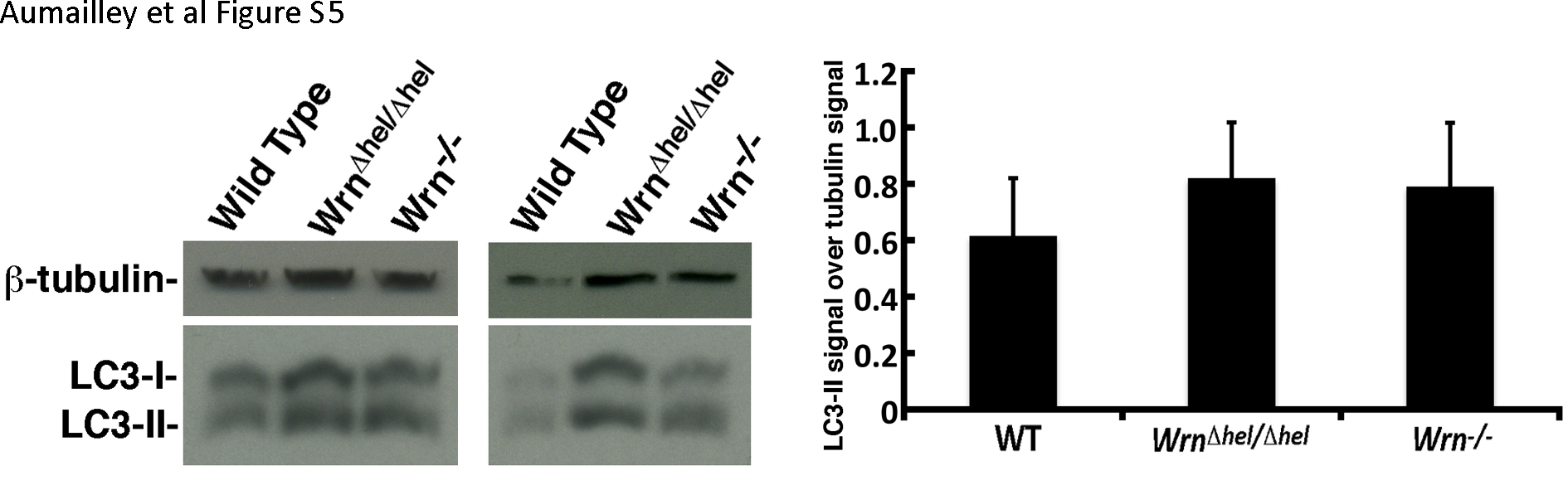

Supplement: S5 Fig — The histogram on the right represents the ratio of LC3-II signal over β-tubulin signal from two independent experiments. (JPG) [file pone.0140292.s005.jpg]

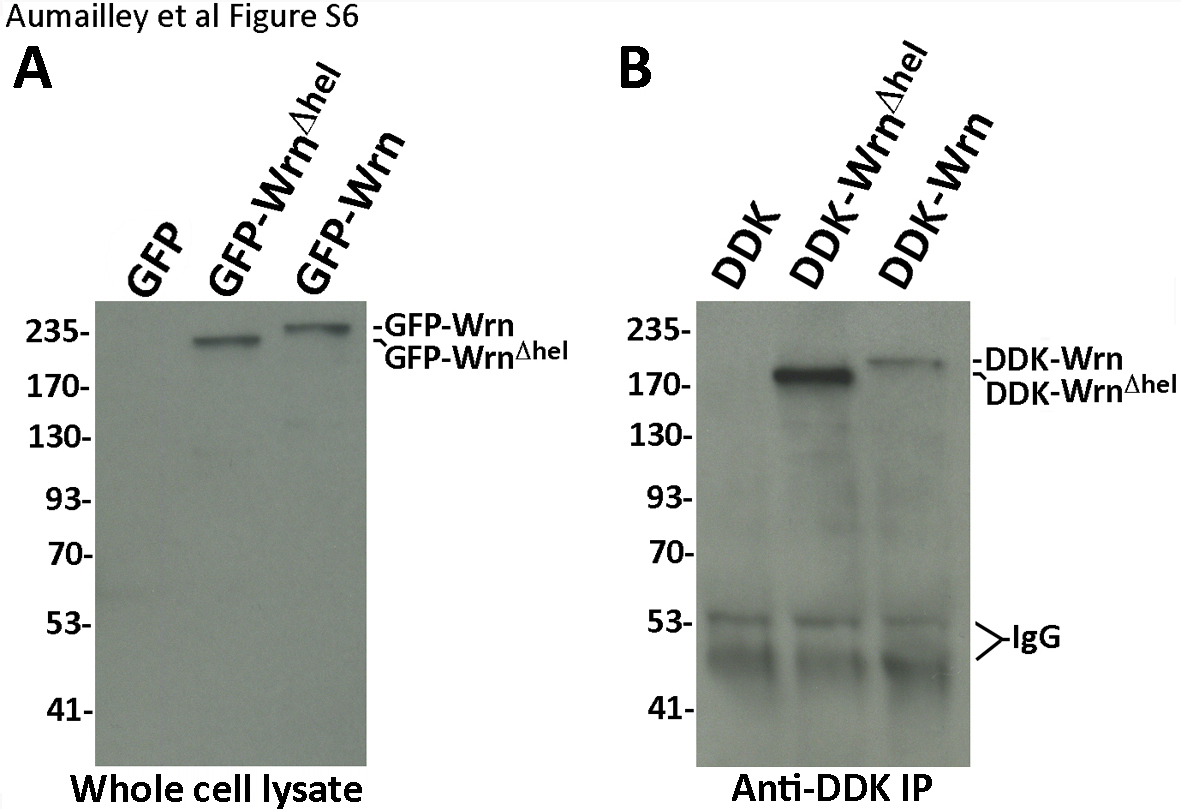

Supplement: S6 Fig — (A) Example of a western blot showing the expression of the WT GFP-Wrn and the mutant GFP-WrnΔhel proteins in total cell lysates. (B) Example of a western blot showing the expression of the immunoprecipitated WT DDK-Wrn and the immunoprecipitated mutant DDK-WrnΔhel proteins. Proteins were immunoprecipitated with the anti-DDK antibody and revealed by western with an antibody against the Wrn protein. (JPG) [file pone.0140292.s006.jpg]

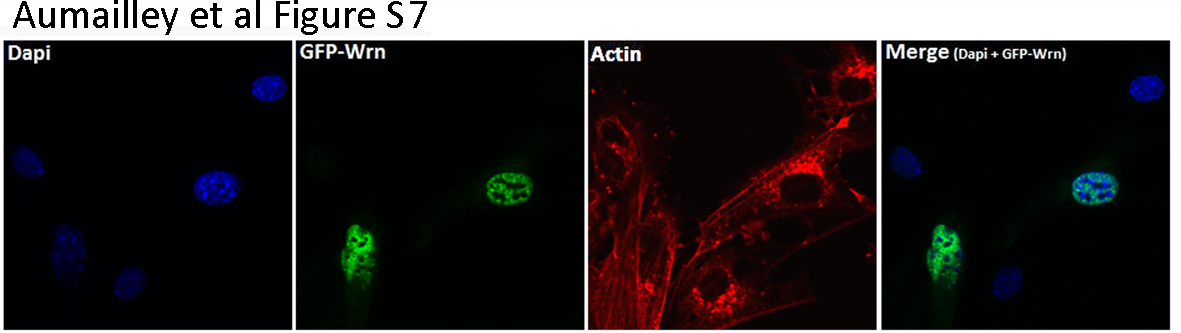

Supplement: S7 Fig — The actin network is revealed by using a fluorescent phalloidin reagent. (JPG) [file pone.0140292.s007.jpg]

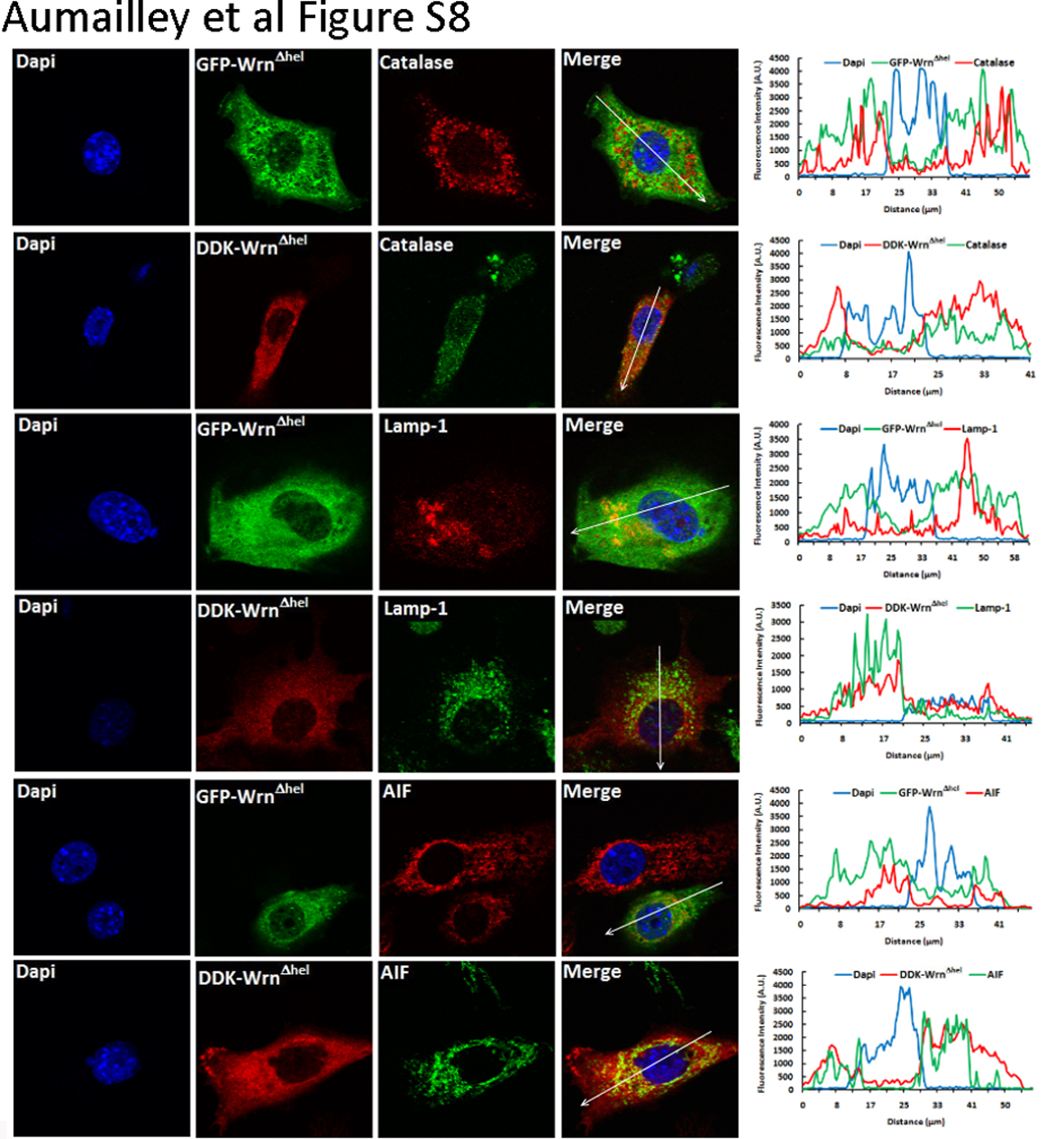

Supplement: S8 Fig — Images in the first row represent the localization of the GFP-WrnΔhel mutant protein and catalase (peroxisomal marker) in Wrn Δhel/Δhel MEFs. Images in the second row represent the localization of the DDK-WrnΔhel protein and catalase in Wrn Δhel/Δhel MEFs. Images in the third row represent the localization of the GFP-WrnΔhel protein and Lamp-1 (lysosomal marker) in Wrn Δhel/Δhel MEFs. Images in the fourth row represent the localization of the DDK-WrnΔhel mutant protein and Lamp-1 in Wrn Δhel/Δhel MEFs. Images in the fifth row represent the localization of the GFP-WrnΔhel mutant protein and AIF (mitochondrial marker) in Wrn Δhel/Δhel MEFs. Images in the sixth row represent the localization of the DDK-WrnΔhel mutant protein and AIF in Wrn Δhel/Δhel MEFs. The graph at the end of each row represents the intensity of the fluorescence along the arrow in the merge image. (JPG) [file pone.0140292.s008.jpg]
